# Abrasion and Erosion Resistance of Cermets: A Review

**DOI:** 10.3390/ma15010069

**Published:** 2021-12-22

**Authors:** Jakob Kübarsepp, Kristjan Juhani, Marek Tarraste

**Affiliations:** Department of Mechanical and Industrial Engineering, Tallinn University of Technology, Ehitajate tee 5, 19086 Tallinn, Estonia; jakob.kubarsepp@taltech.ee (J.K.); marek.tarraste@taltech.ee (M.T.)

**Keywords:** cermet, hardmetal, ceramic–metal composite, wear by hard particles, wear resistance, wear mechanism, abrasion, erosion

## Abstract

WC-based hardmetals are employed widely as wear-resistant ceramic–metal composites for tools and wear parts. Raw materials supply, environmental concerns and some limitations of hardmetals have directed efforts toward development of alternative wear-resistant composites–cermets. We present a current state of knowledge in the field of ceramic-rich (≥50 vol%) cermets behavior in abrasion and erosion conditions, which are the dominant types of wear in many industrial applications. Distinction is made between two-body and three-body abrasion, solid-particle erosion, and slurry erosion. Cermets, in particular TiC-, Ti(C,N)- and Cr_3_C_2_-based composites and hardmetals, are compared for their abrasive and erosive wear performance and mechanism. The review enabled formulation of tribological conditions in which cermets may be comparable or have potential to outperform WC-Co hardmetals. Hardmetals, in general, outperform cermets in abrasion and solid-particle erosion at room and moderate temperatures. However, cermets demonstrate their potential mainly in severe conditions—at elevated temperatures and corrosive (oxidation, electrochemical corrosion) environments.

## 1. Introduction

Losses due to wear and friction comprise approximately 1.4% of the world GDP [1]. In some industries, such as mining, building materials production, recycling of wear parts and consumables may compose a substantial part of life-cycle costs. Managing these costs requires the development of advanced wear-resistant materials and advanced material production technologies.

There is a variety of materials used in wear applications. Engineering ceramics and ceramic–metal composites are being employed as resistant to wear bulk materials in many application fields. However, the lack of availability of wear-resistant materials in bulk form or difficulty or expense in its manufacturing can force the use of coatings instead [2]. The most widely used ceramic–metal composites for wear applications are WC-based hardmetals (cemented carbides). The production of hardmetals is an important growing segment of the powder metallurgy industry [3,4]. However, substitution of critical raw materials (CRM) [5] is and will be essential in the future manufacturing chain. Both W and Co in hardmetals are included in the list of CRM of the EU of 2021. Both metals are of high economic importance and supply risk. Reducing or replacing the CRM is one of the innovation efforts of the hard material industry—development of new hard materials with alternative hard phase (beyond WC) and metallic phase (beyond Co) [3].

Hardmetals comprise the most important sector of the hard and wear-resistant materials industry, but there are many other ceramic-based materials, in particular, cermets. Cermets have been defined in different ways. This paper uses the definition from [6]: cermets are ceramic–metal composites bonded with a metal matrix except for WC-based composites–hardmetals (cemented carbides). Cermets consist primarily of ceramic particles, such as titanium carbonitride (Ti(C,N)) or titanium carbide (TiC), Ti(C,N)- and TiC-based cermets exhibit high hardness, exceeding that of WC-Co hardmetals (at similar volume % of ceramic phase) and resistance to wear at high cutting rates as compared to hardmetals. However, cermets are generally more brittle and less ductile than WC-based hardmetals. The most widely used metallic binders of cermets are Ni alloys [6]. However, during the last two decades, substantial research efforts have been directed to employing Fe alloys as metallic components of cermets. Fe alloys have advantages over Co and Ni, such as high strength, potential to heat treatment and reasonable cost.

In different industrial situations, wear has become a crucial problem. The well-founded selection of appropriate wear-resistant materials is a complicated task that takes into account different types and mechanisms of wear occurring in practice. The knowledge of the acting wear mechanism is essential for a well-founded material selection. Under two- or three-body abrasive wear, for example, prevailing wear mechanism is abrasion while under sliding wear, adhesion and surface fatigue might be predominant [7]. However, considering wear mechanisms, the criteria for the material selection may be different even if the type of wear (e.g., two-body or three-body abrasive wear) seems to be similar [7].

The process of mechanical design may include also the consideration of tribological (wear) performance. However, wear resistance is not merely a property of a material but a property of a tribological system, which makes the prediction of wear resistance difficult [8]. In order to propose wear reducing methods, the designer needs to establish the wear mechanism and also to understand the factors that control wear. The methods for estimating the rates of wear are: (1) wear measurement in an actual operation system in service or (2) wear measurement under the conditions that simulate those in service. Wherever possible, standard testing methods as well as their modifications are recommended for use [9]. For example, for abrasive wear testing, dry sand rubber wheel abrasion test (ASTM G65), high-stress abrasion test (ASTM B611) and wet-sand rubber wheel abrasion test (ASTM G105) are used. Importantly, the results from the laboratory tests should allow us to obtain reliable data for practical use. There are several principal advantages of using standard testing methods [10]. However, as not all of the standard tests used allow such data to be obtained; wear testing in simulative conditions is also used [11,12].

Excellent resistance to abrasion and erosion is one of the primary reasons for selecting such ceramic–metal composites as WC-Co hardmetals for use in a variety of applications. The selection of hardmetals for different wear conditions by hard particles is based on extensive research during several decades. Mechanical properties and wear behavior of WC-Co hardmetals are addressed thoroughly in handbooks covering hard materials [6,13] and friction and wear of materials [2,6,8].

The abrasive wear of WC-Co hardmetals has been widely studied for decades [14,15,16,17,18,19,20,21,22,23,24,25,26,27,28] etc. It is known that abrasion of hardmetals depends primarily on hardness [14,15,16,17] etc. However, there is still some uncertainty as to the effect of changing WC grain size and metallic binder content at constant hardness values as the magnitude of abrasion depends substantially on the microstructure. It was shown that the abrasion resistance of hardmetals could be increased by increasing the grain size of WC while adjusting the Co content to keep the hardness constant [16]. At the same time, some authors show that when the WC grain size becomes very small (submicron), the abrasion resistance increases more than would be expected from the increase in the hardness [18,19]. According to [20], the mapping of properties, such as abrasive wear against microstructural parameters (Co fraction, WC grain size), could be used for the selection of WC-Co hardmetal grades.

The abrasive wear depends to a great extent on abrasion conditions, in particular on the characteristics (hardness, particle size) of the abrasive used. The harder the abrasive, the higher the wear rate. Several authors have proved that at abrasive wear under the same test conditions, the wear increases by almost an order of magnitude when alumina (Al_2_O_3_) abrasive is used instead of silica (SiO_2_) [17,21], etc. The size of abrasive particles also has a significant effect on the wear rate. It has been shown that the use of alumina (Al_2_O_3_) abrasive of different sizes leads to a significant increase in the abrasive rate at the increased size of the abrasive. However, for the silica (SiO_2_) abrasive, the effect of the abrasive particle size was small [17,22], etc. The reason indicated was different friability rates of different abrasives. It should also be taken into account that the magnitude of wear may alter when the size of the abrasive is about the same as the carbide grain size in WC-based composites [19,23]. Abrasion in corrosive media can radically affect the wear behavior–rate of abrasion may be an order of magnitude worse under acidic compared to alkaline conditions [23,24].

Solid-particle erosion of WC-Co hardmetals has been widely studied [22,29,30,31,32,33,34,35], etc. Hardmetals usually do not behave (at least on the microscopic scale) in a classical brittle manner when subjected to a jet of abrasive particles. The combination of material microstructure, properties and erosion test conditions determine the response of a WC-Co composite to an erodent stream [29]. The severity of erosion is imposed by the impingement angle and jet velocity. The nature of an erodent determines as to whether a severe erosion (ductile response domination) or a mild erosion (brittle response domination) regime prevails [6,22]. Large particle sizes, high particle hardness and angularity promote severe wear [6]. In the case of abrasive wear, the dominant solid-particle erosion mechanism depends on the scale of individual particle contacts [6].

It is of particular importance to identify the predominant material removal mechanism for matching an industrial application to a particular hardmetal grade, as the impingement angle at which maximum wear occurs changes with the microstructure [6]. Metallic materials show the maximum wear rate at low impingement angles while brittle materials do at a normal angle of incidence. Because of the two-phase ceramic–metal microstructure, WC-Co hardmaterials demonstrate a more complex response, with the maximum occurring at intermediate impact angles [29]. Hardmetals, similar to both ductile materials (metals) and brittle materials (such as technical ceramics), exhibit the threshold velocity of abrasive jet to cause material loss by the solid-particle erosion [6].

As different from WC-based hardmetals, the wear behavior of cermets in abrasion and solid-particle erosion has been scarcely studied. The reason is that possible cermet compositions and structures appear in a very wide scope. The well-founded selection of an appropriate ceramic–metal composite (hardmetal or cermet) is a challenge. An actual potential of cermets in abrasive wear and erosion is still a question to be answered. The authors of this paper assume that this review is the first attempt to present the current state of knowledge concerning abrasion and erosion of cermets.

**Figure 1:**
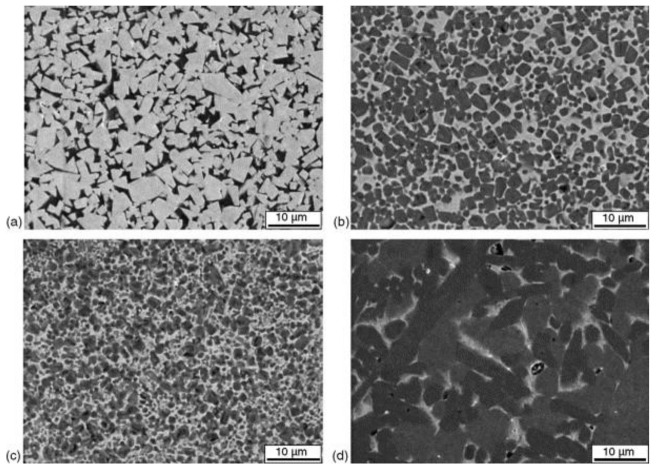
Microstructures of WC-Co hardmetal (**a**) and TiC–FeNi (**b**), TiC–NiMo (**c**) and (**d**) Cr_3_C_2_–NiMo cermets [37].

The present paper aims to summarize the current state of knowledge concerning resistance to abrasion and erosion of cermets. The primary aim is based on the knowledge that wear by hard particles, in particular, abrasive wear and solid-impingement erosion, are widely dominant types of wear, especially in many industrial applications [36]. The present review addresses wear performance of ceramic-rich (≥50 vol% of ceramic phase) cermets, prevalently Ti(C,N)- and TiC- and Cr_3_C_2_-based composites. The typical microstructures of the WC-Co hardmetal, TiC- and Cr_3_C_2_-based cermets are shown in Figure 1. As the microstructure has very strong influence on abrasion and erosion [25], the role of microstructure and mechanical and physical properties in wear are also addressed. Cermets are always compared to WC-Co hardmetals in view of their tribological and mechanical characteristics and in view of performance in service. Therefore, if possible, WC-based hardmetals and cermets are also benchmarked for their tribological performance, provided that the similar tribological system was used. This approach enabled us to formulate tribological conditions in which cermets may be comparable or have potential to outperform WC-Co hardmetals widely used in industry.

## 2. Abrasive Wear

Abrasive wear occurs when hard particles or sometimes hard protuberances on a counterface are forced against and are moved along the surface. The amount of material removed depends on the normal load pressing particles against the surface and the sliding distance. A distinction is usually made between the two-body and the three-body abrasive wear and between low-stress (abrasive particles remain unbroken during abrasion) and high-stress (abrasive particles are broken during the wear process) abrasion [8]. This terminology is used also in the present review.

The wear rate and mechanism depend on the material characteristics, in particular, on the hardness of abrasive particles H_a_ and the wearing material H_m_. The hardness of hardmetals and cermets ranges from 800 up to 2300 HV [6]. Distinction is made between “soft” abrasion (H_a_/H_m_ < 1.2) and “hard” abrasion (H_a_/H_m_ > 1.2) [8,21,26]. During “soft” abrasion, the abrasive particles are not able to indent the surface of a composite, in particular, hardmetal or cermet. However, gradual extrusion and removal of the binder of ceramic–metal composites takes place. Compressive stresses of carbide grains are gradually relaxed, followed by fragmentation of carbide grains [8]. Depending on the ratio H_a_/H_m_, the variation of the wear resistance of different materials in “soft” abrasion conditions is pronounced and depends on the stiffness of the ceramic skeleton (e.g., WC in hardmetals and TiC/Ti(C,N) in cermets) and the mechanical characteristics of the metallic binder. The wear of hardmetals and cermets with hardness over about 900 HV by silica (SiO_2_) particles with hardness of about 1100 HV falls into this category. On the other hand, abrasive wear of ceramic–metal composites with silicon carbide (~2800 HV) or diamond (≥8000 HV) particles can be considered as “hard” abrasion. In the “hard” abrasion regime, abrasive particles can act as cutting tools causing direct plastic deformation of the composite surface. It demonstrates that wear mechanisms in “soft” and “hard” regimes are different. Relative ranking of materials may also be different, depending on the H_a_/H_m_ ratio [8,21,26]. Relative rankings may substantially change at elevated temperatures. The wear by alumina (~2000 HV) particles may be in “soft” or “hard” regime, depending on the surface hardness of the material.

### 2.1. Two-Body Abrasive Wear

Two-body abrasive wear of cermets has been studied in [38,39,40,41,42,43,44,45,46,47,48,49]. Composition, processing technique (hot consolidation conditions), structure (ceramic phase grain size), mechanical properties and two-body abrasive wear testing conditions are summarized in Table 1. Common laboratory tests employ low-stress abrasion testing schemes when the material to be tested is rubbed against a fixed abrasive medium. Related ASTM standards are G132 (test method for pin abrasion testing), G174 (test method for measuring abrasion resistance of materials by the abrasive loop contact), G195 (test method for conducting wear tests using a rotary platform abrasive), and G171 (test method for scratch hardness using a diamond stylus) [50]. Nonstandard tests such as the block-on-ring abrasion test are also used [51]. Testing of hardmetals and cermets has mostly been performed in the “hard” abrasion regime, using SiC and Al_2_O_3_ as abrasives.

Mainly fixed abrasive tests (ASTM G132), single abrasion tests/scratch tests (ASTM G171) and also block-on-ring low-stress abrasion tests (employing adapted ASTM B611 approach) have been used for the study of two-body abrasive wear of cermets [38,39,40,41,42,46,47,48,49]. High-temperature two-body loose abrasive tests of cermets have been performed in [43,44,45]. Results in this section of the paper are presented starting from “hard” followed by “soft” abrasion.

In the pin-on-disc testing scheme, the two-body “hard” abrasion studies of WC-Co hardmetals are more widespread than relevant studies of cermets. The ductile and brittle response of WC-based hardmetals in terms of structure and size of abrasive particles during wear in the “hard” abrasion regime by SiC as abrasive is reported in [18,19]. It was shown that two-body abrasion resistance depends on hardness (determines the penetration of the abrasive into the material) and on the size of the WC grains. However, the effect of WC grain size reduction is larger than that of hardness. The reason is that the carbide grain size influences the fracture and the material removal mechanism, which is related to the homogeneous (ductile) behavior of the nanoscale ceramic–metal composite in contrast to the heterogeneous (brittle) behavior of conventional microsize composites. The transition from the homogeneous to the heterogeneous response depends on the relative sizes of the abrasive particle contact and the hard phase regions in the ceramic–metal composites [8].

Pin-on-disc abrasion tests using a modified ASTM G132 scheme and silicon carbide (SiC) as abrasive were used in [39,41]. (Ti,W)C-Ni-Co-Cr cermets with a binder fraction of 18.5–26.6 vol% using 180 µm SiC grit paper were tested [39]. The focus is on the effect of the Ti/W ratio (1.2, 2.5 and 5) and the Co/Ni ratio (pure Co and Co-50 wt% Ni) on the microstructure and abrasion resistance of this type of cermets. Their results showed that the wear rate has good correlation with the overall cermet hardness, which is strongly influenced by the composition of the binder. Hardness and, as a result, wear resistance of Co-bonded cermets is higher than that of Co/Ni-bonded (50/50). It was also shown that the WC-18 vol% Co hardmetal used as reference material outperforms (Ti,W)C-based cermets irrespective of the Ti:W ratio [39]. The testing of abrasion resistance of TiC-NiMo (Ni:Mo = 3:2) cermets (70, 80 and 90 vol% TiC) using 80 µm SiC grit paper showed that although 90 vol% TiC-NiMo cermet outperformed 80 vol% TiC-NiMo composite in hardness (1664 and 1510 HV, respectively), the wear rate of both composites was similar. The higher fracture toughness of 80% TiC cermets compared to 90% TiC cermets (9.5 MPa m^1/2^ and 7.8 MPa m^1/2^, respectively) is probably the reason for the high wear performance of the 80 vol% TiC-NiMo composite [41].

A special non-standardized two-body abrasive wear tester enabling low-intensity wear at a wide range of temperatures from 20 to 900 °C was used in [43,44,45]. Oxidation abrasion of TiC-NiMo cermets, with a wide range of TiC fraction (40–80 wt%) and three different Ni:Mo ratios (1:1, 2:1 and 4:1) using loose SiC medium with the particle size of 1–2 mm, was performed. No direct correlation between the amount of the metals (Ni, Mo) in the precursor powder or the metallic binder composition (ratio of Ni:Mo) and the high temperature wear rate of the cermets was found. However, materials performance maps constructed facilitate the selection of TiC-NiMo cermets, providing an optimum composition for high temperature applications [43]. It was proved that at high temperatures ≥700 °C, the Cr_3_C_2_-Ni cermets outperform TiC-NiMo composites due to abrasion and oxidation synergy [45].

In the pin-on-disc abrasion test, the ASTM G132 scheme with an aluminum oxide (Al_2_O_3_)-based grinding wheel was used in [47]. Mechanical characteristics and the wear behavior of TiC-Inconel 625 (NiCrMoNb-superalloy) metal matrix composites with different carbide fraction (25, 50 and 70 vol%) were studied. Composites were produced using squeeze casting with the infiltration of matrix (Inconel 625) melt. Hardness and wear performance improved significantly with the addition of 25 vol% TiC. Surprisingly, no further increase in TiC from 50 to 70 vol% resulted in an additional improvement of hardness and abrasive wear resistance [47].

**Figure 2:**
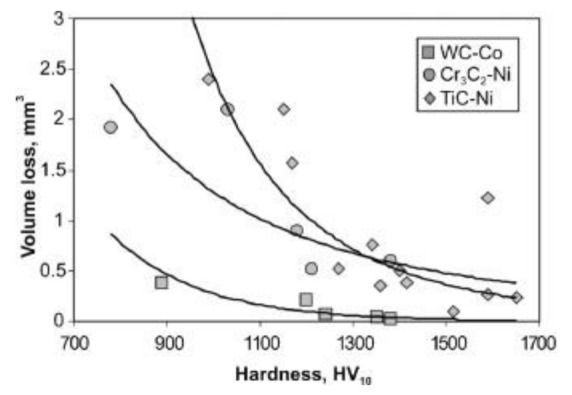
Total wear volume in the two-body dry abrasive wear of the cermets and hardmetals as a function of sliding distance at 20 N load [46].

A similar approach—using Al_2_O_3_ grinding wheel for the low-stress two-body abrasive wear tests—was applied by Pirso et al. [46,48]. However, the adapted testing scheme used was block-on-ring similar to the ASTM B611 standard, replacing the steel wheel with an abrasive grinding wheel. A wide range of different cermets (TiC-NiMo with the binder fraction of 20–60 wt% and Ni:Mo ratio of 1:1, 2:1, and 4:1) and Cr_3_C_2_-Ni (10–30 wt% Ni) were studied. WC-Co hardmetals (6–20 wt% Co) were used as reference ceramic–metal composites. It was shown that abrasive wear resistance of hardmetals and cermets depends on the generic group (family) of ceramic–metal composites (WC-, TiC- and Cr_3_C_2_-based) and carbide/binder ratio (see Figure 2). The specific wear rate (wear coefficient) of WC-Co hardmetals is markedly lower compared with TiC-NiMo and Cr_3_C_2_-Ni cermets at the same binder volume fraction and at the same hardness. Wear resistance of TiC-based cermets decreases with a decrease in the Ni:Mo ratio. It was also shown that coarse-grained WC-20 wt% Co hardmetals outperform medium-grained composites in the abrasive wear conditions used.

Research of the wear mechanism of ceramic–metal composites based on different carbides (TiC, WC, Cr_3_C_2_) showed that abrasive wear mechanisms are similar. Wear mechanism depends mainly on the hardness of the material and the ratio H_a_/H_m_. Since the hardness of the Al_2_O_3_ wheel is higher than that of the cermets and hardmetals, microploughing is the dominant wear mechanism. The wear of low-binder cermets (≤15 vol% binder) is elastic-plastic deformation of the surface, followed by a fracture of large carbide grains and carbide skeleton. In the cermets with a higher binder content (>20 vol%), significant plastic deformation of the surface (ploughing) occurs [46]. While the wear mechanism does not depend on the production technology (conventional PM or reactive carburizing sintering), reactive sintered cermets show higher wear resistance [48]. Higher interphase bond strength and more homogeneous carbide grains distribution are the reasons that improve the performance of reactive sintered cermets. The advantage of reactive sintered materials over conventionally produced composites is more distinctly expressed at higher vol% of metallic binder.

One of the two-body abrasive wear resistance tests in the “hard” abrasion regime is a scratch test (single abrasion test) with a diamond stylus, enabling the evaluation of material resistance to scratching damage. Such tests allow for the comparison of materials relatively easily and in a short period of time, enabling good repeatability. Scratch testing is also a technique to provide more fundamental information on the wear mechanisms [52,53]. A single-scratch test by a conical diamond indenter with 100 nm diameter under 15 N load of TiC-(Fe-Co-Ni-Cr-Mo) cermets (~50 vol% of carbides), prepared by conventional vacuum sintering, was performed in [40]. It was shown that the size of hard ceramic particles and the hardness of materials are two factors for the abrasion resistance of cermets. Larger particle sizes of TiC-based cermets resulted in a narrower width of the scratches as well as better abrasive wear resistance. This result is inconsistent with the results of a previous research of WC-Co cemented carbides/hardmetals (with grain size from nanosize 0.07 to 2.5 µm and hardness of 1100–2300 HV), showing that the nanostructured composites exhibit higher scratch resistance [18]. The scratches are smaller by virtue of higher hardness of nanostructured WC-Co hardmetals.

**Figure 3:**
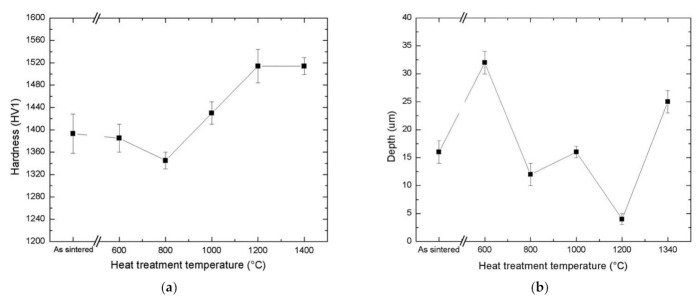
Measured hardness (**a**) and scratch depths (with 30 N applied load) (**b**) as a function of TiC-Ni_3_Al cermet processing conditions (as-sintered and ordering heat treatment at temperatures 600–1340 °C) [38].

Scratch tests with different applied loads of 10–100 N with a Rockwell conical diamond indenter (tip radius of 200 µm) sliding in linear motion across the flat test sample surface were recently used by a research group of Dalhouse University [38,49]. They studied TiC with nickel aluminide binder (TiC-30 vol% Ni_3_Al) cermets produced by in situ reaction sintering of TiC, Ni and Al powders. The effects of postsinter heat treatments (600–1340 °C) on the atomic ordering of the Ni_3_Al were assessed through Vickers indentation and scratch testing. An increase in hardness from 1400 to 1530 HV was observed as a result of ordering heat treatment at 1200 °C (see Figure 3a). As a result, measured scratch depths from the same samples were reduced from ~15 to less than 5 µm (see Figure 3b). A remarkable effect of heat treatment (austenitization followed by aging) on the mechanical characteristics (hardness, fracture toughness) and the scratch resistance of TiC-30 vol% 17–4 PH (AISI Type 630 steel) cermets with a precipitation hardenable stainless steel binder was also shown in [49]. These results indicate substantial influence of the metallic binder structure and properties on the wear performance even at comparatively low volumetric fractions. This influence is in agreement with the conclusions of a previous research of WC-based hardmetals, showing a substantial influence of the regions of relatively soft metallic phase coexisting with harder phases on the abrasive wear resistance [19,26].

“Soft” abrasion (using silica (SiO_2_) as the most common abrasive in industrial applications with the particle size of 0.2–0.3 mm) of a wide range of TiC-NiMo cermets (NiMo fraction of 20–60 wt% and Mo:Ni ratio of 1:1, 1:2 and 1:4) was studied at a wide range of temperatures by Antonov et al. [44]. Abrasive wear tests were performed at 20, 400, 700 and 900 °C. Materials wear performance maps showed the effect of oxidation kinetics on abrasion at different temperatures. The best wear resistance at high temperatures was demonstrated by cermets with a high NiMo binder content (50 and 60 wt%) and high Mo:Ni ratio (high Mo content) in the binder [44].

### 2.2. Three-Body Abrasive Wear

Three-body abrasive wear of cermets was studied in [37,54,55,56,57,58,59,60,61,62,63,64]. Table 2 summarizes the composition, hot consolidation conditions, structure (grain size of ceramic phase), mechanical properties and three-body abrasive wear testing conditions. Common laboratory tests employ low-stress and high-stress testing regimes. Related ASTM standards for low-stress three-body abrasion are G65 (dry-sand rubber wheel abrasion test) and G105 (wet sand rubber wheel abrasion test). For the high-stress abrasion test, ASTM B611 (high-stress abrasion resistance test) [50] or ISO 28,080 (hardmetals: abrasion test for hardmetals) are used. Modifications of standard tests are also widely employed [51]. These rotating wheel abrasive wear tests performed in agreement with standards or using their modified versions have been widely employed in the studies of wear behavior of cermets [37,54,55,56,57,58,59,60,61,62,63,64]. The most common abrasive used in the tests is silica (SiO_2_). Harder abrasives such as SiC and Al_2_O_3_ [54,55,57] and diamond [57] were also employed. In this review paper, results are introduced starting from “hard” followed by “soft” abrasion.

Abrasive wear behavior of the Ti(C,N)-based commercial cermet (Chinese grade FD22, grain size 0.5–2 µm, hardness 2200 HV) was studied using wet sand rubber-rimmed wheel test system and coarse abrasives (particle size about 0.3–0.9 mm) of SiC, Al_2_O_3_ and SiO_2_ [55]. Due to the high hardness of the cermet, only abrasion with SiC (H_a_/H_m_ ≈ 1.12–1.32) may be considered as “hard” abrasion. The wear of the Ti(C,N) cermet increases with the increase in the sliding distance, abrasive mass fraction in slurry (fed into the small space between the wheel and the samples) and hardness of the abrasive. The abrasive wear mechanism of the cermet mainly depends on the relative hardness between the cermet and abrasives, H_a_/H_m_. In “hard” abrasion conditions with SiC abrasive microcutting, grain fracture and plastic deformation with grooves were found the dominant wear mechanisms. When Al_2_O_3_ was used, plastic deformation and ploughing grooves were the main wear mechanisms. During “soft” abrasion with SiO_2_ extrusion, and removal of the binder phase and slight plastic deformation with grooves were the dominant wear mechanisms. The same research group studied also three-body abrasive wear resistance in the same testing conditions of the WC-8 wt% Co hardmetal [27]. Interestingly, while the hardness of the WC-Co hardmetal compared unfavorably with the hardness of the Ti(C,N)-based cermet (1500 HV vs. 2200 HV), the hardmetal outperformed the cermet in “hard” abrasion conditions when SiC and Al_2_O_3_ were used as abrasives. However, in “soft” abrasion conditions with SiO_2,_ the cermet outperformed the hardmetal due to higher hardness [55].

**Figure 4:**
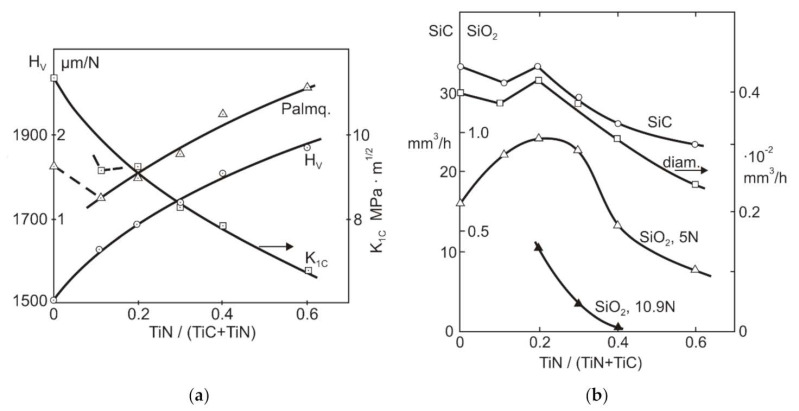
The Vickers hardness and Palmquist cracking susceptibility (µm/N), bulk fracture toughness K_IC_ (**a**) and abrasion wear rate (**b**) vs. alloy ratio TiN/(TiN + TiC) [57].

Abrasive wear of TiC-NiMo cermets with TiN–to–(TiN + TiC) ratios between 0 and 0.6 was tested by Larsen-Basse [57]. Cermets sintered with a binder of 12.5 wt% Ni–11 wt% Mo had 10 wt% VC addition. The composites were abraded under three-body conditions using a steel wheel and SiC, SiO_2_ loose abrasives and 1 µm diamond polish paste (see Table 2). The three abrasives all gave the highest wear rates (lowest wear resistance) for intermediate values of alloy hardness and toughness—at a TiN/(TiN + TiC) ratio of 0.2. The lowest wear rate for the hardest alloy was shown at an alloy ratio of 0.6 (see Figure 4). It should be noted that while the grain size of most specimens was around 1 µm, for alloys with TiN/(TiN + TiC) ratios of 0.2 and 0.3 (showing the greatest wear), the grain size was 3–4 µm. This difference had no clear effect on the mechanical properties but could possibly be responsible for the lowest wear resistance of coarse-grained composites [57].

It was shown in [57] that the wear mechanisms for the larger abrasives (SiC, SiO_2_) are similar to those described for WC-Co hardmetals, i.e., plastic indentation and microspalling for the hard abrasive (SiC) and fine-scale microspall formation for the relatively soft abrasive (SiO_2_). The diamond polish gave the same “hard” wear mechanism as the SiC abrasive but on a smaller scale. Abrasion by SiO_2_ is highly load and specimen hardness dependent—excess load and increase in material hardness favor abrasive crushing and change in the wear mechanism.

Sintering technology influences the structure formation processes and, as a result, the mechanical and wear performance. Three-body abrasive wear of TiC-50 wt% high manganese steel (13 wt% Mn, 2 wt% Cr, 1.1 wt% C) cermets produced using different hot consolidation processes (vacuum sintering, hot pressing (HP), microwave sintering (MS) and spark plasma sintering (SPS)) was studied by a research group of University of Science and Technology Beijing [54]. SiC with a particle size of about 0.25 µm was used as abrasive. The samples consolidated by microwave sintering demonstrated the best wear resistance. It was concluded that the high hardness and transverse rupture strength are the reasons behind the good wear resistance of MS cermets.

In the laboratory tests, the conditions employed should be relevant to the real life conditions. The three-body abrasion of cermets using SiO_2_ as an abrasive is described in [37,56,58,59,60,61,62,63,64]. A research group of Tallinn University of Technology addressed the behavior of vacuum sintered cermets and WC-Co hardmetals in three-body abrasive wear conditions [37,58,59,60,61,62,63,64]. Two different block-on-ring testing procedures were used: (1) modification of ASTM B611 standard applying water slurry of SiO_2_ (particle size of 0.1–0.3 mm) [58,59,61,64] or (2) ASTM G65 dry sand rubber wheel test with a similar abrasive [37,60,62,63].

Three-body abrasive wear of a wide range of TiC-NiMo cermets (40–80 wt% TiC, Ni:Mo ratios of 4:1, 2:1 and 1:1) using two loads (40 N and 200 N) is reported in [61]. Hardmetals WC-Co was used as the reference composite (see Table 2). The range of mechanical properties of the tested materials was considerable: hardness 810 HV_10_ (at 40 wt% TiC) up to 1650 HV_10_ (at 80 wt% TiC), transverse rupture strength (TRS) 730–2450 N/mm^2^ and fracture toughness K_IC_ 10.4 MPa m^1/2^ as minimum. At the low load of 40 N, cermets with 20 wt% NiMo (low-stress abrasion) and at the high load of 200 N (high-stress abrasion), cermets with 40 wt% NiMo demonstrated the highest wear resistance. In both cases, the lowest wear rate was observed at the Ni:Mo ratio of 1:1. However, taking into account higher mechanical characteristics (TRS, K_IC_), alloys with the Ni:Mo = 2:1 ratio are recommended for use as wear-resistant structural materials. For comparison, at equal hardness, the wear rate of the WC-Co hardmetal was found substantially lower than that of TiC-NiMo cermets [61].

**Figure 5:**
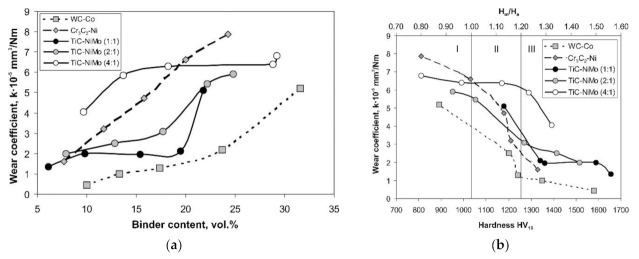
Wear coefficients vs. binder content (**a**) and bulk hardness (**b**) of TiC- and Cr_3_C_2_-cermets, and WC-Co hardmetals [59].

Three-body abrasive wear of TiC-NiMo cermets, the same grades as in [61] and additionally, of Cr_3_C_2_-Ni cermets (10–30 wt% Ni) and range of WC-Co hardmetals (6–20 wt% Co), were studied under low-stress (40 N) and high-stress (200 N) conditions [59]. Although enhancing the hardness of a particular material usually leads to a decrease in the wear rate, hardness is not a good prediction of the relative wear resistance of materials of different types (families). It was also shown that the abrasive wear resistance depends on the generic group (family) of ceramic–metal composites (WC-, TiC- and Cr_3_C_2_-based) and their carbide/binder ratio and can differ at equal binder fraction and hardness by several times (see Figure 5). The lowest wear coefficient (wear rate) was demonstrated by the WC-Co hardmetals. However, similar to two-body abrasion three-body abrasive wear mechanism of different ceramic–metal composites is similar and depends on the ratio of H_a_/H_m_ and loading conditions. The authors suggest that abrasive wear behavior (wear mechanism) can be divided to three zones according to the material/abrasive ratio H_m_/H_a_. In zone I (H_m_/H_a_ < 1, i.e., “hard” abrasion regime) and in zone III (H_m_/H_a_ > 1.2, i.e., “soft” abrasion regime), the wear rate is in weak dependence of the hardness. In zone II (H_m_/H_a_ = 1–1.2), the abrasive wear rate depends considerably on the hardness of the composites (see Figure 5b).

**Figure 6:**
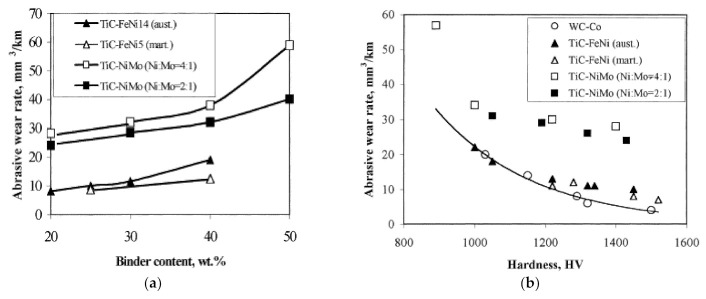
Abrasive wear rate of TiC-NiMo and TiC-FeNi cermets vs. binder content, NiMo ratio and structure (**a**) and Vickers hardness (**b**) [64].

Research on Ni- and Co-free cermets, in particular, TiC- or Ti(C,N)-Fe alloy composites, has been intensified markedly during the last two decades. As a result, the diversity of cermets has contributed substantially to the problems in material selection. As an example, studies have focused on high-stress three-body abrasive wear behavior of TiC-FeNi (60–80 wt% TiC, different Ni contents 5–17% and structure of binder) and TiC-NiMo (50–80 wt% TiC, Ni:Mo ratio of 4:1 and 2:1) cermets and WC-Co hardmetals (80–90 wt% WC) [64]. All the vacuum sintered composites were of medium grain size of 1.0–2.2 µm (WC-Co hardmetals) and 1.9–2.2 µm (TiC-based cermets) (see Table 2). It was shown that in high-stress abrasion conditions, TiC-based cermets with a suitable composition and structure of the binder, in particular, the FeNi binder, can compete with WC-Co hardmetals at equal hardness (see Figure 6).

**Figure 7:**
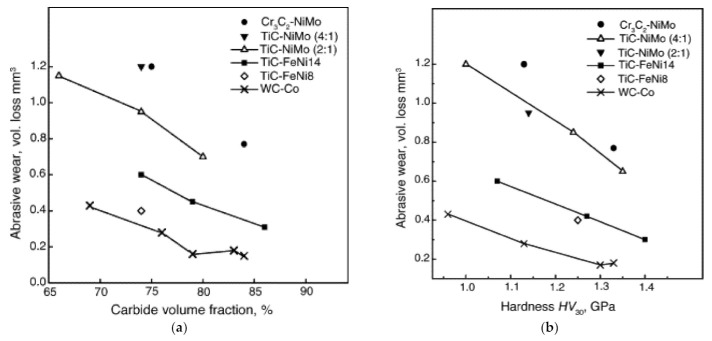
Three-body abrasive wear of TiC-based cermets and WC-Co hardmetals vs. carbide volume fraction (**a**) and Vickers hardness (**b**) [37].

Wear performance of pressureless vacuum-sintered TiC-FeNi, TiC-NiMo cermets and WC-Co hardmetals was also compared by the ASTM G65 dry-sand rubber wheel abrasion testing scheme [37,62]. At equal carbide volume fraction and hardness, WC-based composites are at an advantage over TiC-based cermets. Comparing cermets at room temperature, TiC-FeNi cermets outperform TiC-NiMo composites (see Figure 7). It is evident from Figure 7 that while the prognosis of abrasive wear resistance on the basis of hardness can lead to pronounced mistakes, there is correlation between the wear performance and the hardness within each group (family) of ceramic–metal composites. It was suggested that the resistance to abrasive wear depends, first of all, on the fraction and properties of its carbide phase (modulus of elasticity E) and second, on those of the metallic binder (proof stress in compression R_C0.1_). The higher abrasive wear resistance (at room temperature) of TiC-FeNi cermets compared to TiC-NiMo (Ni:Mo ratio of 4:1 and 2:1) composites may result from the higher strength properties (proof stress) of TiC-FeNi cermets, in particular, composites with martensitic structure of a binder [37]. A significant effect of the strength of the metallic binder on the abrasive wear resistance of WC-based hardmetals was also reported by Larsen-Basse [21,26]. It was shown that an FeNi alloy with higher strength than Co gives greater wear resistance for the same mean free path of the metallic binder both in “soft” (SiO_2_ as an abrasive) and “hard” (SiC as an abrasive) abrasion conditions. Further increase in the binder strength by heat treatment results in the further increase in the wear resistance.

High abrasive wear performance of the high strength Fe alloy bonded cermet (in particular, iron-aluminide bonded TiC-FeAl) comparable to that of the WC-Co hardmetal (at similar vol% of carbides) was demonstrated in [56]. In terms of production technology, as compared to the pressureless vacuum sintering, pressure-assisted sinter/HIP consolidation technology enables the reduction of porosity of TiC-FeNi cermets and enhancement of the resistance to brittle failure. At the same time, consolidation technology has no effect on the abrasive wear and solid-particle erosion resistance [63].

**Figure 8:**
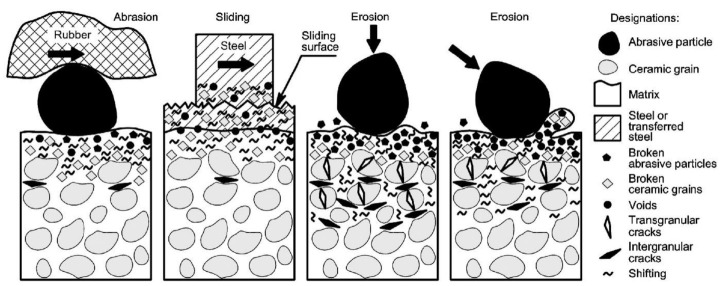
Features of cermet surface transformation under abrasive, sliding and erosive wear [60].

It has been shown that the formation of subsurface mechanically mixed layers (MML) is an essential feature of carbide composite response to the applied loading during abrasive, erosive and sliding wear. Below MML, there is a region that contains inter- and transgranular cracks located just below the surface while intergranular cracks were revealed at a distance of about 30 µm below the surface (see Figure 8). Transgranular cracks are very rare under three-body abrasive wear conditions. The number of fine broken ceramic grains in Cr_3_C_2_-Ni and TiC-NiMo cermets and WC-Co hardmetals was high after abrasion under high contact pressure. The knowledge about the microstructure and composition of the subsurface layer can assist more reliable estimation of the wear resistance as compared to the surface hardness value [60].

The wear mechanism of WC-Co hardmetals and cermets may be somewhat different taking into account mechanical properties, in particular, toughness of the ceramic phase used. It was found that contribution to the wear of hardmetals came from the removal of the binder phase from the surface layers and accumulation of plastic deformation in the WC grains, followed by fracture and fragmentation. Although subsurface cracking may contribute to material loss, it is not thought to be a dominant mechanism of abrasive wear and erosion of WC-Co hardmetals [28]. However, it may be an important mechanism for cermets. Studies of WC-based hardmetals and TiC-NiMo cermets showed that unlike hardmetals, for cermets brittle microfracture may be the dominant wear mechanism [21,26].

Within the studied broad array of TiC- and Cr_3_C_2_-based cermets with different binder fraction and composition, it is necessary to address the effect of ceramic phase grain size. It has rarely been addressed in the wear behavior studies of cermets. However, for WC-Co hardmetals, high structure sensitivity has been demonstrated by several researchers. It has been shown that hardness can be used as an indirect measure of abrasion resistance only at low hardness values, i.e., when the wear process occurs predominantly by means of plastic deformation. At higher hardness values, i.e., when the microfracture plays an important role in the wear mechanism, abrasion resistance depends substantially on the carbide grain size. Grades of equal hardness but different grain size have, in general, different wear resistance. Coarse grades have higher abrasion resistance in the 1000–1600 HV hardness range, while finer grades are expected to have higher abrasion resistance at hardness values higher than 1600 HV [15,16]. Studies of structure (ceramic phase grain size and distribution) on the sensitivity of the behavior of cermet abrasive wear are needed in the future.

### 2.3. Summary

#### 2.3.1. Two-Body Abrasive Wear

Two-body abrasion of TiC-, (Ti,W)C- and Cr_3_C_2_-based cermets with predominantly Ni alloy binders has been studied by ASTM G132, ASTM G171 and non-standard block-on-ring low-stress abrasive wear testing schemes. Research has been conducted in the “hard” abrasion (Al_2_O_3_ or SiC, diamond abrasives) and “soft” abrasion (SiO_2_) regimes, at room and elevated temperatures up to 900 °C.

It was shown that the abrasion rate, in general, has good correlation with the overall ceramic–metal composite hardness, which is strongly influenced by the fraction, composition, structure and properties of the metallic phase. Substantial effect of metallic binder characteristics is observed even at comparatively low volumetric fractions.

The two-body abrasive wear mechanisms of cermets and WC-based hardmetals are similar. Wear mechanism depends mainly on the hardness of the material and the ratio H_a_/H_m_. However, the wear performance of cermets and hardmetals depends also on the generic group (family) of composites (TiC-, Cr_3_C_2_- or WC-based). At room temperatures, WC-Co hardmetals outperform TiC-based cermets, while TiC-based cermets outperform Cr_3_C_2_-based at the same level of hardness or binder volumetric fraction. At high temperatures (≥700 °C), Cr_3_C_2_-based cermets compare favorably with TiC-based ceramic–metal composites due to synergy of oxidation and abrasion.

#### 2.3.2. Three-Body Abrasive Wear

In the three-body abrasion studies of TiC-, Ti(C,N)- and Cr_3_C_2_-based cermets with Ni- and Fe alloy binders, low-stress abrasion (ASTM G65, ASTM G105) and high-stress abrasion (ASTM B611) regimes or their modifications have been used.

Three-body abrasive wear mechanism of cermets and WC-Co hardmetals are, is general, similar and depend mainly on the ratio H_a_/H_m_ and the loading conditions. However, in high-stress abrasion conditions, the wear mechanism may differ to some extent, taking into account properties, in particular, strength and toughness of ceramic phase and domination of brittle microfracture during the abrasion of cermets.

Hardness is not a property that allows for good estimation of the wear resistance if materials of different families are considered. Wear depends on the generic group (family) and the ceramic/binder ratio of a composite. The three-body abrasive wear depends, first of all, on the fraction and properties of the ceramic phase (WC vs. TiC or Cr_3_C_2_) and second, on those of the metallic binder.

WC-Co hardmetals outperform (at room temperature) cermets in “hard” abrasion conditions—at similar hardness, the wear rate of hardmetals is substantially lower than that of cermets. In the “soft” abrasion conditions, cermets, in particular those bonded with iron alloys, may be comparable to hardmetals upon conditions of higher hardness. At room temperature, TiC-Fe alloy cermets outperform cermets bonded with nickel alloy. The higher abrasive wear resistance of Fe alloy bonded cermets may result from the higher strength properties of Fe alloys, in particular heat-treatable grades.

## 3. Erosive Wear

Solid-particle erosion occurs when discrete solid particles strike a surface. It differs from three-body abrasion primarily in the origin of forces between the particles and the wearing surface. In erosion, the extent of wear depends on the number and mass of individual particles striking the surface and on their impact velocity [8]. The difference of erosion from the abrasive wear lies in its fluid contribution to the mechanical action producing material removal. Solid-particle erosion is common in any system in which a gas stream carries abrasive particles. If loose abrasive particles are carried by a liquid, the wear is termed as slurry erosion [8,51]. The present paper also uses this terminology when addressing erosion of cermets.

Hardmetals and other ceramic–metal composites (cermets) do not always behave in a classical brittle manner as engineering ceramics when subjected to an erosive fluid jet. At a microscopic scale, they can show some attributes of both ductile and brittle behavior [6,8]. Factors determining the severity of erosion are jet velocity, impingement angle and the nature of the erodent (particle size, hardness and angularity). Similar to abrasive wear, erosion mechanism can involve prevalently plastic deformation (ductile behavior) or brittle fracture (brittle behavior). The domination of these behaviors depends on the scale of the abrasive particle impact zone relative to the microstructure (grain size) of a ceramic–metal composite. In the ductile mode, erosion of the material removal occurs by the plastic flow and fracture of the binder. It is a dominant erosion mechanism when the number of WC grains encompassed in the impact zone exceeds ~100 (i.e., homogeneous response). The effects of microstructure of the composite on the erosion in the ductile mode are mainly through the hardness. In the brittle mode, the material removal occurred mainly by cracking and crushing of WC grains. It is a dominant erosion mechanism when the number of carbide grains encompassed in impact is small, ~10 (heterogeneous response) [8,30,31,32]. The effects of the microstructure of the ceramic–metal composite on the erosion in the brittle mode are more complex; in addition to hardness, fracture toughness and particle size of erodents are involved. However, if the abrasive particles are very small, with their size comparable to the structural constituents (grains), wear can occur by preferential erosion of the metallic binder phase, leading to undercutting and removal of intact ceramic grains. Severity of erosion of WC-Co hardmetals is low and ductile mechanism prevails [8].

### 3.1. Solid-Particle Erosion

Solid-particle erosion of cermets has been considered in [33,34,37,48,58,60,64,65,66,67,68,69,70,71,72,73,74,75,76,77,78,79,80,81,82,83,84,85,86,87]. Composition, hot consolidation conditions, structure (grain size of ceramic phase), mechanical properties and testing conditions of solid-particle erosion are summarized in Table 3. Commonly used methods for erosion testing can be divided into two: those in which abrasive particles are accelerated in gas (or liquid stream) and those with circular motion used to achieve the impact velocity. The standard methods employ a gas-blast scheme. The common laboratory test is ASTM G76 (test method for conducting erosion by solid-particle impingement), which uses a stream of high pressure gas to accelerate a stream of abrasive (Al_2_O_3_) particles through a nozzle toward a test sample. Gas-blast procedure for testing at elevated temperatures (600 °C) is ASTM G211 [50]. Modifications of these testing procedures (e.g., using silicon carbide (SiC) or silica (SiO_2_) as an abrasive) are used. Centrifugal accelerator of abrasive particles for erosion testing at room and elevated temperatures is widely employed [8,12,51].

Solid-particle erosion tests of cermets have been prevalently performed using four-channel centrifugal accelerator where up to 15 specimens can be eroded under identical conditions [34,37,48,58,60,64,65,70,71,72,73,74,75,76,77,78,79,80,81,82,83,84,85,86]. Gas-blast testing procedures (ASTM G76, ASTM G211) have not been so widely used [66,67,68,69,87]. High-temperature erosion of cermets and also hardmetals has been studied in [60,66,71,73,87]. Research results in present paper are presented starting from “hard” erosion followed by “soft” erosion.

Severity of erosion, similar to abrasion, depends on the hardness of abrasive H_a_ and wearing material H_m_. Depending on the H_a_/H_m_ ratio, distinction should be made between “hard” and “soft” erosion. Erosion rate of hardmetals and cermets with SiC particles exceeds, as expected, by a factor of about 10 erosion rate with silica (SiO_2_) abrasive [72,75,76,81,83,84].

**Figure 9:**
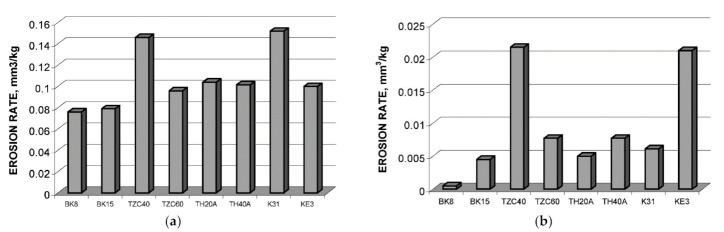
Steady state erosion rates of ceramic–metal composites: abrasives (**a**) SiC (0.1–0.3 mm) and (**b**) SiO_2_ (0.1–0.3 mm). Erosion conditions: jet velocity 61 m/s, impact angle α = 75°. Materials: TZC40, TZC60 (TiC-40 and 60 wt% FeCrSi, respectively); TH20A, TH40A (TiC-20 and 40 NiMo (Ni:Mo ratio 2:1), respectively); K31, KE3 (Cr_3_C_2_-15 and 30 wt% Ni, respectively); BK8, BK15 (WC-8 and 15 wt% Co, respectively) [83].

As abrasive particles are significantly harder than the surface (“hard” erosion), the erosion (similar to abrasion) generally demonstrates relatively low variation. The wear is highly sensitive to the structure and mechanical characteristics (in particular hardness) of a material surface when H_a_/H_m_ is about 1 (“soft” erosion) [8]. This expectation was clearly confirmed in the study of solid-particle erosion of ceramic–metal composites (hardmetals and TiC- and Cr_3_C_2_-based cermets), using both silicon carbide and silica as abrasives (see Figure 9).

**Figure 10:**
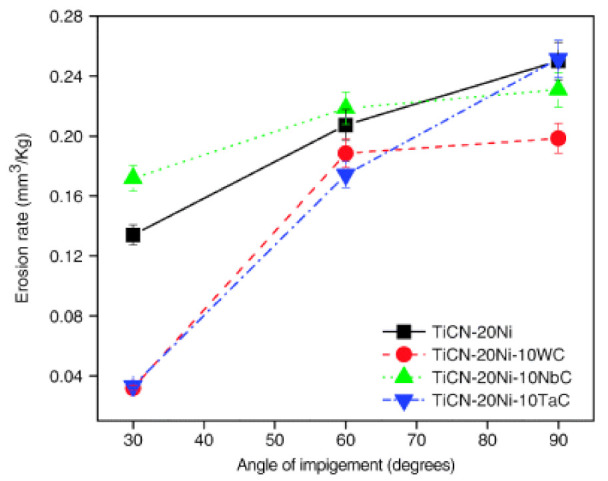
Erosion rate vs. impact angle for Ti(C_0.7_N_0.3_)-based cermets (erodent SiC with particle size ~66 µm, mass flow rate 2.33 g/s) [67].

Erosion wear behavior using SiC as an abrasive has been reported in [67,72,74,75,76,77,81,83,84]. Erosion of Ti(C,N)-20 wt% Ni cermets containing 10 wt% secondary carbides WC/NbC/TaC was studied in [67]. The Ti(C_0.7_N_0.3_)-based cermets were vacuum-sintered (see Table 3). The erosion rate was observed to increase with an increase in the impingement angle (30°→90°). This relationship between the erosion rate and the impact angle follows a trend similar to brittle ceramic materials (see Figure 10). WC additives, unlike NbC, TaC, Mo_2_C, resulted in an increase in the erosion resistance of Ti(C,N)-20Ni cermet under all investigated angles of impingement.

Erosion rate of brittle materials depends on hardness and fracture toughness [8,30,31,32] and can be generally expressed as

(1) $$E=C*K_{IC}^{m}*H_{m}^{n}$$

where *C* is a constant depending on the wear conditions and *K_IC_* is material fracture toughness and *H_m_* is a wearing material hardness. Different exponents *m* and *n* are proposed in [8,88,89], but in all models, the role of fracture toughness of brittle materials is dominant, i.e., |*m*| > |*n*|. In [67], the model with *m* = −1.3 and *n* = −0.25 is applied. It is concluded that no relationship exists between the erosion rate (at normal impact) and the parameter $K_{IC}^{-1.3}H_{m}^{-0.25}$ for the investigated Ti(C_0.7_N_0.3_)-20Ni cermets. This implies that unlike ceramics, the brittle lateral fracture may not be a dominant mechanism of material removal in the erosion of ceramic–metal composites.

**Figure 11:**
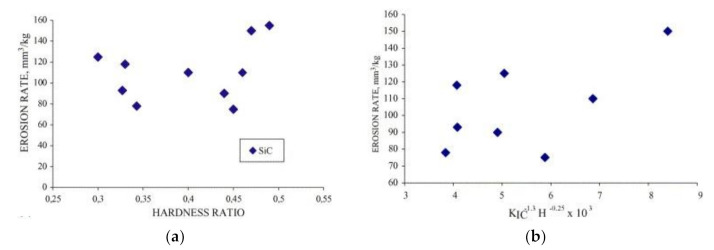
Erosion rate vs. hardness ratio *H_m_/H_a_* (**a**) and *K_IC_^−1.3^H_m_^−0.25^* (**b**) for the WC-, TiC- and Cr_3_C_2_-based composites (erodent SiC 0.1–0.3 mm, particles velocity 60 m/s, attack angle α = 75°) [76].

Erosion of ceramic–metal composites of different compositions (WC-Co, TiC- and Cr_3_C_2_-based cermets) was studied by the research group of Hussainova [72,75,76,77,81,82,83,84]. Similar to the work in [67], it was also shown that there is no consistent correlation between the erosion rate and $K_{IC}^{-1.3}H_{m}^{-0.25}$ (see Figure 11b) [72,75,76,77,81]. In addition, hardness seems not to enable erosion resistance prediction if composites of different generic families (WC-, TiC- and Cr_3_C_2_-based) are compared (see Figure 11a).

It was suggested that the behavior of non-homogeneous composites (cermets and hardmetals) cannot be evaluated by looking at one mechanical characteristic only or by looking at a blend of the bulk properties. The microstructure (grain size and the strength of interphase bond) and the physical properties of a composite (in particular, thermal conductivity) and phases of a composite (coefficient of thermal expansion) determine the behavior under erosion conditions [72,74,75,76,77,81]. It was concluded that the materials with low difference in the coefficient of thermal expansion of phases and high thermal conductivity are preferable [72,74,76,77].

**Figure 12:**
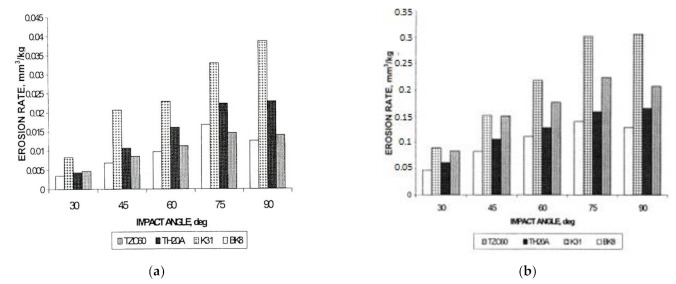
Comparative evaluation of the erosion rate of ceramic–metal composites at different impact angles and velocities: (**a**) 31 m/s; (**b**) 80 m/s. Erodent SiC (0.1–0.3 mm). Materials: TZC60 (TiC-40 wt% FeCrSi); TH20A (TiC-20 wt% NiMo (Ni:Mo = 2)); K31 (Cr_3_C_2_-15 wt%Ni); and BK8 (WC-8 wt%Co) [84].

Ceramic–metal composites (hardmetals, cermets) consist of a high volume fraction of hard ceramic phase and a more ductile metallic binder. Such composites are not necessarily brittle but may be characterized by substantial fracture toughness (in general, 10–25 MPa mm^1/2^), in contrast to most ceramic materials. Their response to erosion is more complex. It is known that brittle and ductile materials respond differently to the angle of impact—ductile materials show commonly peak erosion at a shallow impact angle while brittle materials often show maximum wear for normal incidence [8]. The maximum position depends on material response to impact [75,82,83,84]. While the maximum wear rate occurred for WC-Co hardmetals at an impact angle around 60°, for TiC-based cermets, the maximum is at the impact angle around 75° and for Cr_3_C_2_-Ni cermets, at an angle of 90° (see Figure 12).

Erosive wear behavior of Ti(C,N)-CoNi (~15 vol%) cermets with additions of TaC, NbC, WC and Mo_2_C for cutting tools with different Ti(C,N) fraction (25.8–58.8 vol%) was studied by D’Errico et al. [68,69]. Tests were performed in the conditions comparable to ASTM G76 standard using Al_2_O_3_ (mean particle size of ~70 µm) as abrasive and impact angles of 20° and 90° (see Table 3). It was concluded that the most important controlling factor of cermets is Ti(C,N) content. Antiwear properties of cermets eroded by solid-particle impingement are mainly driven by mechanical properties of composites. Hardness is the most important controlling factor both under oblique (20°) and orthogonal (90°) impacts. Hardness in combination with toughness plays an important role under orthogonal impact [68,69].

The resistance of TiC-20 vol% FeAl, TiB_2_-60 vol% FeAl and WC-20 vol% FeAl composites to solid-particle erosion with Al_2_O_3_ (particle size of 50 µm) was evaluated in a wide range of temperatures (25, 180, 500 and 700 °C) and compared to the erosion behavior of WC-6 wt% Co hardmetals (see Table 3). The impingement angle was 75°, and the duration of each test was 20 min in the nitrogen atmosphere of commercial purity. While at low temperatures (<500 °C), TiC-FeAl cermets compare unfavorably with WC-based hardmetals, this material might be a promising candidate for elevated temperature (>500 °C) applications once the microstructure of cermets is optimized for erosion resistance [87].

Erosion resistance of TiC-Fe cermets (670 HV) produced by combustion synthesis (SHS technology) in conditions similar to ASTM G76 (using Al_2_O_3_ as abrasive, impact angles of 90° and 30°) was assessed at room (20 °C) and elevated (350 °C) temperatures (conditions encountered for combustion boilers). Studies showed that cermets present prevalently brittle erosion mechanism at both temperatures [66]. A detailed study of surface damage during particle-wall collision by the solid Al_2_O_3_ particles (with average particle size of 90 µm) of WC-15 wt% Co hardmetal and TiC-FeSi cermets (40–60 wt% binder) was performed [85]. Ceramic–metal composite targets were impacted with abrasive particles over the range of impact velocities 7–50 m/s at the impact angle 67°. Laser doppler anemometer (LDA) measuring technique was employed for measuring the ratio of the normal component of particle velocity after and before impact with the target—the restitution coefficient (characterizing loss of kinetic energy of particles). Values of restitution coefficients have a good fit with the experimental data of the erosion rate of ceramic–metal composites—the highest restitution coefficient was demonstrated by the WC-Co hardmetal [85]. Level of energy consumption during application seems to be an appropriate guide for material selection in the conditions of erosive wear [72].

Erosion of cermets and hardmetals by “soft” silica (SiO_2_) sand has been addressed in several works [33,34,37,48,58,60,64,65,70,71,72,73,75,76,78,79,80,81,82,83,84,86]. Researchers of University of California [33] studied the erosion of WC-Co and WC-Ni hardmetals and TiC-NiMo cermets at room temperature. All specimens were eroded by quarts abrasive (75–200 µm) at a range of impact angles (30°, 60°, 90°) at 60 m/s in a gas-blast-type rig (see Table 3). It was shown that control of the erosion behavior is transferred from the binder to the carbide skeleton at around 80 vol% carbide. At high binder levels (<80 vol% TiC), the binder controls the erosion but is severely constrained by the carbides and therefore behaves in a brittle manner—maximum erosion occurs at the impact angle of 90°. At lower binder levels (>80 vol% TiC), carbide dominates the eroded surface and the erosion vs. the impact angle plot reflects grain-by-grain removal mechanism, i.e., maximum erosion occurs at intermediate impact angles (~60°). The authors also show that the contiguity of the carbide skeleton is of greater importance than the mechanical properties of particular carbides. Fine carbide grain size and a hard binder should be combined to achieve outstanding erosion resistance. However, results demonstrate clearly that TiC-NiMo cermets are at a disadvantage in relation to WC-based hardmetals at a similar vol% of carbides [33].

Erosion of TiC- and Cr_3_C_2_-based cermets and WC-Co hardmetals with different fractions of carbides and binder compositions has been studied by research groups of Tallinn University of Technology [34,37,48,58,60,64,70,71,72,73,74,75,76,77,78,79,80,81,82,83,84,85,86]. A four-channel centrifugal accelerator allowing the testing of 15 specimens simultaneously (materials examined at the identical erosion conditions) was employed. Tests were carried out at room and also at elevated temperatures (up to 650 °C) using silica (SiO_2_) with the particle size of 0.1–0.3 mm as an abrasive (see Table 3). It is known that in terms of material response to erosion and mechanical properties, ceramic–metal composites are macroscopically brittle but at microscopic level they have mixed ductile-brittle response [8]. Structural parameters (binder vol% and carbide grain size) are decisive effect on mechanical properties (in particular, hardness and toughness) and wear resistance. It is also known that ceramic–metal composites, in particular, WC-Co hardmetals with fine and submicron structure show better solid-particle erosion resistance than medium- and coarse-grained ones [22,28,29,31,32]. In the studies of TiC-based cermets and WC-Co hardmetals (used as reference materials) [34,37,48,58,60,64,70,71,72,73,74,75,76,77,78,79,80,81,82,83,84,85,86], commonly medium-grained composites (average grain size 2–2.7 µm) were used, while Cr_3_C_2_-based cermets were mainly coarse-grained (average grain size > 3 µm).

The mechanically mixed layer (MML) is developed in the cermets subjected to wear (see Figure 8). The comparison of tribolayer formation during abrasive and erosive wear showed that intergranular cracks were formed at the depth up to 30 µm. Intergranular and transgranular cracks are more easily formed in ceramic–metal composites with high carbide content. The stiff response of the surface enhances the crushing of the abrasive particles as well. Deeper cracks are formed at a concentrated impact and higher energy–erosion under normal angle of impact and high velocity of 80 m/s. Transgranular cracks found under conditions of three-body abrasive wear were rear [60].

**Figure 13:**
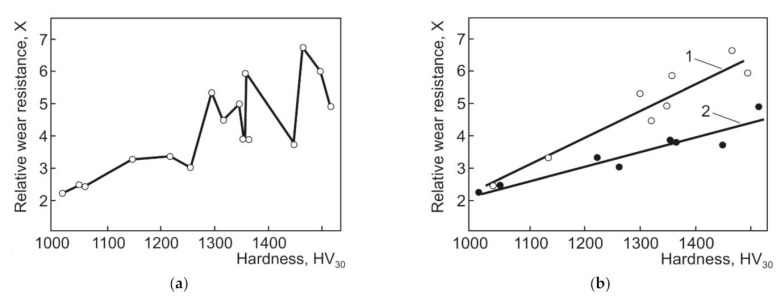
Relative erosive wear resistance vs. hardness of TiC-based cermets with different vol% carbides and composition, structure and properties of the binder: (**a**)–all cermets; (**b**) cermets with distinguished binder families, 1—martensitic steel binder, 2—austenitic steel binder and Ni:Mo (4:1) binder (abrasive SiO_2_, impact velocity 80 m/s, attack angle α = 30°) [34].

The erosive wear resistance of TiC- and WC-based composites with a wide range of carbide content (80–90 wt% WC in WC-Co hardmetals and 40–80 wt% TiC in TiC-FeSi, TiC-FeCrSi, TiC-FeNi, TiC-FeCrNi and TiC-NiMo cermets) was studied in [34]. It was shown that prognosis of erosive (and abrasive) wear resistance on the basis of single mechanical properties, in particular hardness, results in mistakes when carbide composites of different families (chemical composition and structure) are considered (see Figure 13a). However, considerable differences in the structure and hardness of the metallic binder cause differences in the gradients of the relative wear resistance functions X = f(HV) (see Figure 13b).

**Figure 14:**
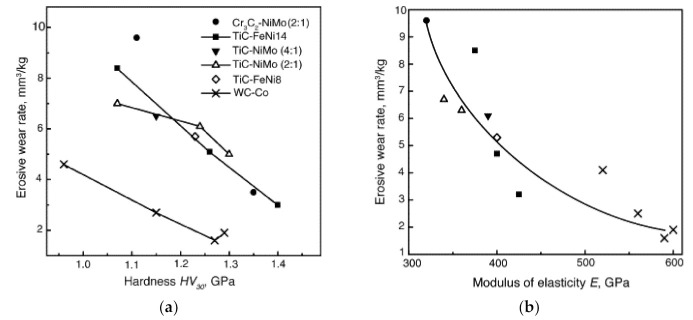
Erosion rate vs. hardness HV (**a**) and modulus of elasticity E (**b**) of different families of ceramic–metal composites: TiC- and Cr_3_C_2_- based cermets and WC-Co hardmetals (abrasive SiO_2_, impact velocity 80 m/s, attack angle α = 30°) [37].

Hardness can be used as the first approximation for the assessment of erosion resistance also within each group of ceramic–metal composites based on different carbides characterized by different physical and mechanical properties. All carbide composites show clearly decreasing erosion rate with increasing hardness (see Figure 14a). At equal hardness (and also carbide vol%), WC-Co hardmetals are at an advantage over TiC- and Cr_3_C_2_-based cermets (at room temperatures) [37].

The performance of carbide composites in erosive (and abrasive) wear is controlled by the stiffness of the material—its resistance to the elastic (evaluated by the modulus of elasticity E) and plastic (evaluated by the proof stress in compression R_C0.1_) strains and depends primarily on the carbide phase (its fraction and grain size) and secondly on the composition, structure and properties of the binder. The modulus of elasticity as a measure of material stiffness may be used as the first approximation for the evaluation of the “soft” erosion resistance of ceramic–metal composites independent of the ceramic phase used [34,37,72,76,78,83,84] (see Figure 14b). A better correlation exists between erosion resistance and rigidity characteristics of cermets and hardmetals using a combined characteristic, including both the modulus of elasticity and proof stress in compression– $E^{2}R_{C0.1}$ [64,79].

In the study of the effect of microstructure on the erosive wear of cermets, Hussainova and Antonov [72,76,80] concluded that the relative ranking of different cermets with respect to the erosion rate could be explained first of all by the microstructures and thermomechanical properties of composites whereas hardness or fracture toughness seems to be of minor importance. However, modulus of elasticity may be used for the evaluation of erosion resistance in the conditions of mild wear. Analysis of the cermet grain size and the erosion rate showed relationships similar to those for WC-Co hardmetals. It seems that there is a threshold carbide size. Exceeding the threshold results in changing the wear (fracture) mechanism from intercarbide of intracarbide failure [72]. A similar approach has been proposed for WC-Co hardmetals—between 1.6 and 2.2 µm, there must be a critical grain size above which WC grains deform (fracture) at relatively low stress [29].

High-temperature erosion of carbide composites was studied in [60,71,73]. At room temperature, WC-Co hardmetals outperform TiC- and Cr_3_C_2_-based cermets (at the same vol% of binder and/or hardness) (see Figure 14). Testing of WC-Co hardmetals, Cr_3_C_2_-Ni and TiC-NiMo cermets (all with 12 vol% binder) showed that at 600 °C, TiC-NiMo cermet outperforms WC-Co hardmetal and Cr_3_C_2_-Ni cermet at both impact angles (30°, 90°) and abrasive jet velocities (20 and 80 m/s) (see Table 4). It was shown that erosive behavior of composites possessing similar binder contents (12 vol%), grain sizes and mechanical properties can be explained on the basis of formation and fracture of a mechanically mixed layer (MML). WC-12 vol% Co has the lowest erosion resistance and the thickest MML, consisting of oxides and a damaged layer of bulk material. TiC-12 vol% NiMo material showed the highest erosion resistance and MML formed at its surface is less pronounced [73]. Thickness, structure and properties of tribolayer (MML) influence erosive wear behavior also at room temperature [60].

**Figure 15:**
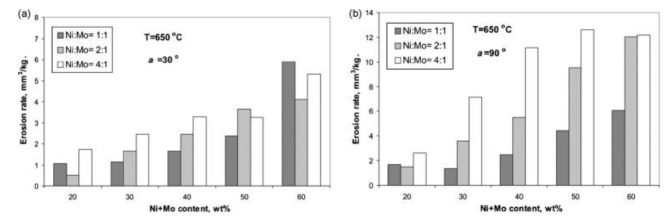
Erosion rate of TiC-NiMo cermets at 650 °C vs. NiMo content, NiMo ratio and impact angle of (**a**) 30° and (**b**) 90° (SiO_2_ abrasive jet velocity 50 m/s) [71].

Further research was focused on the high-temperature erosion of TiC-NiMo cermets of different binder fractions (20–60 wt%) and Ni:Mo ratios (4:1, 2:1, 1:1) of the binder [71]. It was shown that high-temperature erosion resistance of cermets increases with a decrease in the binder content (increase in TiC fraction) and increase in the Mo content of the binder (see Figure 15).

### 3.2. Slurry Erosion

Slurry erosion of cermets has been addressed in [90,91,92,93,94,95,96,97,98,99,100,101,102,103,104,105]. Composition, sintering conditions, structure (ceramic phase grain size), mechanical properties and testing conditions of slurry erosion are summarized in Table 5. A laboratory test commonly recommended is ASTM G75 (determination of slurry abrasivity (Miller number) and slurry abrasion response of materials). ASTM G119 (determining synergism between wear and corrosion) enables one to identify the abrasion and corrosion components by potentiodynamic polarization techniques. In addition, nonstandardized erosion testing techniques, such as the slurry-jet impingement test and the slurry-pot test (propeller test), are used. In the slurry-pot tests, a rotor carrying specimens is immersed in a tank containing slurry of a liquid and abrasive particles [8,51]. In the slurry-jet impingement test, an ejector nozzle is employed to entrain sand particles from a sand bed into a stream of water to form a slurry [104,105,106]. Testing conditions applied for the study of cermet behavior in the slurry erosion (see Table 5) show that slurry-pot tests were most widely used.

Cermets on the base of chromium carbide (Cr_3_C_2_) have some unique properties that make them useful in special applications, such as high-temperature and corrosive environments, and in situations that require high corrosion-abrasion resistance [58]. M. Antonov et al. [104,105] studied erosion-corrosion of Cr_3_C_2_-Ni cermets in the slurry of artificial seawater and SiO_2_ (particle size ~0.1 mm, concentration 6, 8, 10 wt%). The slurry-jet impingement test rig was used, based on the design by Zu et al. [106]. The study focused on the effect of the cermet binder content (10, 20 and 40 wt% Ni), surface roughness (R_a_ = 300–3250 A) together with test conditions, such as abrasive particles concentration, applied potential (−600 mV for cathodic protection, 0, +250 and +500 mV), temperature (20 and 42 °C), and time of experiments (6–120 min) on the performance of cermets. Material wastage, synergy and regime maps were developed, and it was demonstrated that material loss during simultaneous effect of corrosion and erosion is complicated and cannot be evaluated as a simple summation of these processes [104,105].

Slurry erosion of TiC-FeCr cermets (TiC fraction 33, 40, 50 and 60 wt% at different Cr contents in binder) was studied in [103]. WC-Co hardmetal (15% Co), structural carbon steel (0.45% C) and stainless martensitic and austenitic steels were used as reference materials. Erosion tests in that study were performed employing slurry-pot test equipment. The erosion-corrosion environment was tap water—abrasive (SiO_2_, particles 0.3–0.4 mm) slurry with abrasive concentration of 5 wt%. Rotor with specimens peripheral speed was 5.5 m/s, testing time 24 h. Additionally, slurry erosion tests in alkaline conditions (using 0.5% NaNO_2_ as inhibitor) were performed. It was shown that corrosion resistance exerts a dominant effect on the slurry erosion resistance of ceramic–metal composites, in particular, TiC-FeCr cermets and WC-Co hardmetal. As a result of selectivity of corrosion and erosion of these structurally heterogeneous composites, erosion resistance in the water-SiO_2_ slurry may be even to a great extent disadvantageous in relation to non-corrosion-resistant carbon structural steels. In such conditions, corrosion-resistant straight chromium TiC-FeCr cermets outperformed substantially WC-Co hardmetals. Erosion resistance depends on hardness only in the conditions of sufficient corrosion resistance of the composite or provided that corrosion inhibition is used [103].

A series of slurry erosion tests of Ti(C,N)-based cermets were recently performed by the research group of Sichuan University [90,91,92,93,94,95,96,97,98,99,100,101,102]. Slurry-pot test apparatus employed was similar to that used in [103]—samples were fixed on the impellers to rotate in a tank of slurry of a liquid and abrasive particles. Erosion behavior of Ti(C,N)-based cermets was assessed, and the effect of the composition (C/N ratio in a carbonitride, Ni/Co ratio in a binder), addition of carbides (Mo_2_C, Cr_3_C_2_, WC, TaC, and NbC) and test parameters (characteristics of erodents (Al_2_O_3_, SiO_2_), impingement velocity, fluid composition and viscosity) on the degradation resistance of cermets was studied. Neutral [90,91,93,94,96,97], saline (artificial seawater or NaCl solution) [92,94,95,101], alkaline [96,100] and acid [91,98,99,102] slurries of Al_2_O_3_ (particle size 100–250 µm) [90,91,92,93,95,96,97,98,99,100,102] or SiO_2_ (particle size of 100–300 µm) [94,101] were employed. Common abrasive concentration was 5 wt% with an exception of work [91].

**Figure 16:**
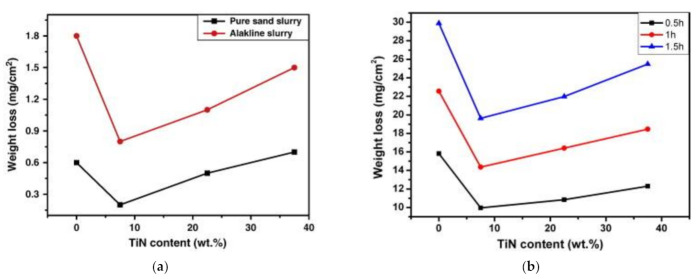
Weight loss of Ti(C,N)-15 wt% Ni cermets in different environments: (**a**) distilled water (neutral) and alkaline (5% NaOH) slurry, testing time 1 h [96]; (**b**) acid (0.5 mol/L H_2_SO_4_) slurry [99].

An increase in the TiN fraction in carbonitride favors grain size reduction of Ti(C,N)-based cermets [96,99]. In distilled water-Al_2_O_3_ slurry, erosion resistance improved substantially with TiN addition (see Figure 16a). In alkaline and acid slurries, the weight loss was produced by the synergistic effect of erosion and corrosion (see Figure 16a,b).

TiC_0.7_N_0.3_-based cermets behavior in saline slurries based on NaCl solution or seawater was studied in [92,94,95,101]. A study of the effect of Mo_2_C/WC ratio in Ti(C,N)-10 wt% Mo_2_C/WC-15 wt% Ni cermets showed that the cermets with the Mo_2_C/WC ratio of 1 demonstrate the best resistance to erosion [95]. Substantial improvement of erosion resistance is possible with the addition of Co to Ni binder, which can be attributed to the decrease in porosity and better solid solution strengthening effect [94].

**Figure 17:**
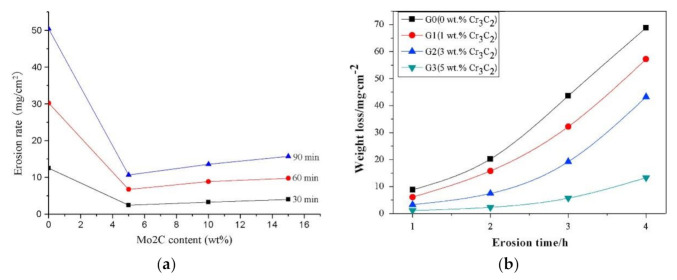
Erosion rate of TiC_0_._7_N_0.3_- 15 wt% Ni cermets as the function of Mo_2_C content (**a**) [102] and Cr_3_C_2_ addition to TiC_0.7_N_0.3_-10 wt% Mo_2_C-15 wt% Ni cermets (**b**) [98].

Research on TiC_0.7_N_0.3_-based cermets behavior in acid slurries (small H_2_SO_4_ additions (0.1 mol/L or 0.5 mol/L) used) was conducted in [91,98,99,102]. It was shown that Mo_2_C additions reduce the Ti(C,N) grain size and can dramatically improve the erosion-corrosion resistance (see Figure 17a) [102]. Erosion resistance of TiC_0.7_N_0.3_-10 wt% Mo_2_C-15 wt% Ni cermets was additionally improved by Cr_3_C_2_ additions, increasing the corrosion resistance of the Ni binder (see Figure 17b) [98]. Additions of Cr_3_C_2_ enhanced slurry erosion resistance also in alkaline slurry circumstances [100].

The erosion corrosion degradation of cermets may be classified to the corrosion regime, erosion-affected corrosion regime, corrosion-affected erosion regime and erosion regime. The contributions of corrosion, erosion and synergy to the erosion-corrosion degradation are strongly environment dependent. With an increase in the environmental corrosivity, the contributions of corrosion and synergy are enhanced considerably [91,98,100].

### 3.3. Summary

#### 3.3.1. Solid-Particle Erosion

In the solid-particle erosion studies of TiC-, Ti(C,N)- and Cr_3_C_2_-based cermets with Ni- and Fe alloy binders, predominantly centrifugal acceleration of abrasive particles has been employed. Gas-blast testing schemes (ASTM G76, ASTM G211) have not been so widely used.

It has been shown that the severity of erosion, similar to abrasion, depends on the ratio H_a_/H_m_. The wear is highly sensitive to the structure and hardness of composites when H_a_/H_m_ is about 1 (“soft” erosion), while in “hard” erosion conditions (H_a_/H_m_ > 1.2), erosion wear demonstrates relatively low variation.

“Hard” erosion rate increases with an increase in the impingement angle: 30°→90° similar to brittle ceramic materials. However, the equations describing correlation between erosion and values of hardness (HV) and fracture toughness (K_IC_) developed for the assessment of the brittle materials erosion rate are not relevant for cermets and hardmetals. These ceramic–metal composites demonstrating substantial fracture toughness may be brittle at a macroscopic level, but at microscopic level, they demonstrate mixed ductile-brittle response.

The response of ceramic–metals composites to the impact by abrasive particles depends on the family (generic group) of composites. While the maximum wear rate for WC-Co hardmetals occurred at impact angles around 60°, for TiC- and Cr_3_C_2_-based cermets, the maximum is at impact angles 75° and 90°, respectively. At room and elevated temperatures (<500 °C), WC-based hardmetals outperform cermets while at high temperatures, cermets, in particular TiC-based composites, are promising erosion-resistant material candidates.

In “soft” erosion conditions, relative ranking of different ceramic–metal composites with respect to erosion rate could be explained first of all by the microstructure (fraction and grain size of carbide), whereas hardness (HV) or fracture toughness (K_IC_) seemed to be of minor importance. However, hardness can be used as the first approximation for the assessment of erosion resistance within each group of ceramic–metal composites (WC-, TiC- or Cr_3_C_2_-based), and modulus of elasticity if ceramic–metal composites of different groups (families) are considered.

The erosion performance of ceramic–metal composites with prevalent fraction of ceramic phase is controlled to a significant extent by the stiffness of the material—its resistance to the elastic and plastic strains depending primarily on the nature of the ceramic constituent and, secondly, on the composition, structure and properties of the metallic binder.

Exceeding the threshold carbide size (around 2 µm) in ceramic–metal composites—cermets and hardmetals—may result in changing the erosion mechanism, resulting in a substantial increase in the erosion rate.

At room and moderately elevated temperatures, cermets are at a disadvantage in relation to WC-based hardmetals at a similar hardness or vol% of carbides. However, at high temperatures (>600 °C), TiC-NiMo cermets outperform both WC-Co hardmetals and Cr_3_C_2_-Ni cermets.

#### 3.3.2. Slurry Erosion

In the slurry erosion studies of TiC-, Ti(C,N)- and Cr_3_C_2_-based cermets, a nonstandard slurry-pot testing scheme has been most widely used. Neutral (tap or distilled water), saline (seawater or NaCl solution), alkaline and acid slurries of Al_2_O_3_ or SiO_2_ were employed.

The material loss during simultaneous effect of corrosion and erosion is complicated and cannot be evaluated as a simple summation of processes. The degradation of ceramic–metal composites is strongly environment dependent and may be classified to the corrosion regime, erosion-affected corrosion or corrosion-affected erosion regime and erosion regime. With the help of the alloying of the ceramic phase (e.g., C/N ratio of Ti(C,N), additions of Cr_3_C_2_, Mo_2_C, WC) and metallic constituent (e.g., addition of Co to Ni or Cr to Fe binder) may enable substantial improvement of slurry erosion resistance of cermets. Corrosion-resistant cermets may substantially outperform straight WC-Co hardmetals even in water-abrasive slurries.

## 4. Concluding Remarks

WC-Co hardmetals/cemented carbides have been employed widely as wear-resistant ceramic–metal composites for tools and wear parts almost a century. At present, hardmetals comprise the most important sector of the hard and wear-resistant materials industry. As both W and Co in hardmetals are of high economic importance and supply risk, also other ceramic–metal composites with an alternative hard phase (beyond WC) and metallic binder (beyond Co) have been developed. Ti(C,N)- and TiC-based cermets are the most well known. However, the wear behavior of cermets in abrasion and solid-particle erosion has not been studied as widely and systematically as the wear behavior of WC-based hardmetals.

The aim of the review was to present a current state of knowledge in the field of wear behavior of cermets. The focus was on abrasion and solid-impingement erosion as widely dominant types of wear in many industrial applications. Cermets are frequently compared to WC-based hardmetals in view of the mechanical and tribological performance. Therefore, hardmetals and cermets were benchmarked for their abrasive and erosive wear performance and mechanism if such comparable experimental data were available and similar tribological systems were employed. It enabled us to formulate tribological conditions in which cermets may be comparable or have potential to outperform WC-Co hardmetals.

The review is focused on the wear performance of ceramic-rich (≥50 vol% of hard phase) cermets. In terms of abrasive wear, a distinction has been made between the two-body abrasive wear (abrasive particles are fixed on the surface of the opposite body) and the three-body abrasive wear (wear is produced by loose abrasive particles between contacting surfaces). In the discussion of erosive wear, a distinction has been made between solid-particle erosion (abrasive particles carried by a gas) and slurry erosion (hard particles carried by a liquid).

Study of cermets behavior in the two-body abrasion regime has mostly been performed in “hard” abrasion regime using SiC and Al_2_O_3_ as abrasives and diamond indenter or stylus in scratch tests. Wear rate, in general, correlates with overall hardness of ceramic–metals composites based on similar hard phase (carbides, carbonitrides) and/or binder composition and structure. A response to the abrasive wear is different for different generic groups (families) of composites. At room temperature, WC-Co hardmetals outperform (at similar hardness) TiC- and Cr_3_C_2_-based cermets. However, at elevated temperatures, due to synergy of oxidation and abrasion, WC-Co composites compare unfavorably with TiC-NiMo and Cr_3_C_2_-Ni cermets. At very high temperatures ≥700 °C, Cr_3_C_2_-Ni cermets show the best two-body abrasion resistance.

In the studies of cermets behavior in three-body abrasion conditions, the most common abrasive used has been silica (SiO_2_), i.e., abrasion has been performed in “soft” abrasion conditions. However, harder abrasives (SiC, Al_2_O_3_) have been also employed. Three-body abrasive wear behavior mainly depends on the relative hardness of abrasive H_a_ and the wearing material H_m_, H_a/_H_m_. Similar to two-body abrasion, the composites’ response to abrasion is different for different families of ceramic–metal composites. The performance is controlled primarily by the stiffness of the material—its resistance to the elastic (assessed by the modulus of elasticity) and plastic (assessed by the proof stress) strains and depends primarily on the properties of a carbide phase (and carbide skeleton) and secondly on the structure and properties of a binder. In general, WC-Co hardmetals demonstrate the lowest three-body abrasion rate (at room temperature). Unlike WC-based hardmetals, brittle microfracture may be the dominant wear mechanism of cermets. However, cermets bonded with high-strength Fe alloys (steels) can compete (at similar hardness) with hardmetals and outperform TiC-NiMo and Cr_3_C_2_-Ni cermets at specific abrasion conditions, in particular, at room temperature.

With regard to solid-particle erosion (similar to abrasion), erosion rate depends on the ratio H_a_/H_m_ and is substantially (by a factor of about 10) higher in “hard” erosion (e.g., SiC as an abrasive) as compared to “soft” erosion (SiO_2_ as an abrasive) conditions. Wear is substantially more sensitive to the structure and mechanical characteristics of the material surface when H_a_/H_m_ is around 1 (“soft” erosion). The severity of erosion is, in addition to abrasive characteristics, influenced by the jet velocity and the impingement angle. The response of ceramic–metal composites to the attack angle is different for materials of different families. While the maximum wear rate occurs for WC-Co hardmetals at the impact angle of around 60°, for TiC- and Cr_3_C_2_-based cermets, depending on the composition, the angle is around 75–90°. At high ceramic phase fraction (>80 vol%), carbide skeleton controls and at lower fraction (<80 vol%), the binder controls the erosion. Structural parameters (binder vol%, ceramic grain size) are decisive factors of mechanical properties as well as erosion resistance and mechanism. Similar to abrasion, hardness can be used as the first approximation for the assessment of solid-particle erosion behavior within each group of ceramic–metal composites based on different ceramic phase (WC, TiC, Cr_3_C_2_, etc.) and/or metallic binder (Ni-, Co-, Fe alloy) characterized by different physical and mechanical properties. In terms of mechanical properties, the solid-particle erosion performance can be assessed by the stiffness of the composite—its resistance to elastic and plastic strain evaluated by the elastic modulus and proof stress, respectively. At equal hardness (and carbide vol%), WC-based hardmetals (characterized by high elastic modulus, toughness, and thermal conductivity) are at an advantage over cermets (at room temperature). However, at high temperatures (≥600 °C), TiC-NiMo cermets outperform hardmetals (also TiC-steel cermets) due to the synergy of oxidation and abrasion resistance and better ability of Ni alloys to handle high working temperatures.

Regarding the slurry erosion, the erosion-corrosion degradation of ceramic–metal composites may be classified (depending on environment) to the corrosion, erosion-affected corrosion, corrosion-affected erosion, and erosion regime. With increasing environment corrosivity, the contribution of corrosion to erosion-corrosion synergy is enhancing considerably. As a result of selective corrosion and erosion of structurally heterogeneous composites, erosion resistance depends on the material’s mechanical characteristics, in particular, on hardness, only on condition of sufficient corrosion resistance.

The selection of wear-resistant material for tool and/or structural component must take into account, in addition to the wear performance, also working conditions (temperature, corrosion and nature of mechanical loads), machinability, etc. Regarding the performance of cermets as resistant to abrasion and solid-particle erosion materials, the following advantages and disadvantages (if compared to “straight” WC-Co hardmetals) can be highlighted:

Advantages

- High resistance to wear by hard particles in severe conditions—at high temperatures (>500 °C) and corrosive (oxidation, electrochemical corrosion) environments;
- High hardness, in particular that of TiC- and Ti(C,N)-based cermets (at similar vol% of ceramic phase) and high resistance to oxidation;
- Substantially lower density and high strength-to-weight ratio, which is critical in specific application conditions;
- Comprising mainly elements (Ti, Cr, Ni, Fe), which unlike W and Co, are not commonly included in the list of critical raw materials (CRM).

Disadvantages

- Lower resistance to wear by hard particles at low (<500 °C) temperatures and in non-corrosive (neutral) environments. However, cermets may have potential (in mild wear conditions) to be on par with WC-Co hardmetals;
- Lower modulus of elasticity, thermal shock resistance, strength (transverse rupture strength) and physical (thermal conductivity) properties. Comparatively low thermal conductivity may be a challenge in machining (in particular grinding) of cermet tools and structural parts;
- Lower bonding strength of cermets to steels using brazing as the main joining method of ceramic–metal and ceramic composites to metals.

Major sources of industrial wear problems in terms of economical significance are through solid-particle erosion, followed by two- and three-body abrasion [36]. However, it is noticeable that very small fraction of research papers mention any applications relevant to the wear testing conditions described in the papers. Although laboratory testing provides data prevalently on the wear behavior not fully relevant to the real-life application conditions, authors of the present review attempt to give recommendations for the selection of ceramic–metal composites (cermets, hardmetals) under different working conditions (see Table 6).

## Figures and Tables

**Table 1:** Summary of composition, processing, structural and mechanical characteristics and two-body abrasive wear testing conditions of cermets.

Composition *	Processing **	Structure ***	Mechanical Characteristics		Wear Testing Conditions ******	Key Observations	Ref.
			Hardness ****	Toughness *****			
(Ti,W)C/18.5–26.6 vol% (Ni, Co, Cr)	Sinter/HIP (1474 °C)	-	950–1300	-	ASTM G132, pin-on-disc, 180 µm SiC grit paper, F = 4.7 N	WC/18 vol% Co outperforms cermets at similar hardness	[39]
TiC/10–30 vol% (Ni, Mo) (Ni:Mo = 3:2)	LPS (1500 °C)	-	1405–1664	7.8–9.5	ASTM G132/DIN50330, pin-on-disc, 80 µm SiC grit paper, F = 29.4 N	The highest wear performance of 10–20 vol% NiMo cermets	[41]
TiC/25, 50, 75 vol% Inconel (NiCrMoNb superalloy)	Squeeze casting + MI	Wide range of d_TiC_ = 1.0–19.0	75–80 HRA	-	Modified ASTM G132, pin-on-disc, (Al_2_O_3_ grinding wheel), F = 5.08 N	Improvement of wear resistance with TiC fraction increase	[47]
TiC_0.87_/50 (Ni, Mo) (Mo 0 … 8.5 wt%)	SHS	d_TiC_ = 1.9–8.64	-	-	Modified ASTM G132 (conditions not specified)	Mo decreased wear rate due to interfacial bonding strength	[42]
TiC/30 vol% Ni_3_Al	LPS (1550 °C) + heat treatment	d_TiC_ = 2.8–4.4	1400–1530	14.0–18.5	ASTM G171, Rockwell diamond indenter, F = 30 N	Scratch resistance improvement using heat treatment	[38]
TiC/70 (Fe, Co, Ni, Cr, Mo) + Cr_3_C_2_, Mo_2_C	LPS (1200–1370 °C)	-	64.4–67.6 HRC	TRS 1514–2358 MPa as maximum	ASTM G171, conical diamond indenter, D = 100 µm, F = 15 N	The larger d_TiC_ results in higher abrasion resistance	[40]
TiC/30 vol% 17–4PH precipitation hardenable stainless steel	LPS (1550 °C + heat treatment)	-	1159–2342	14.3–19.4	ASTM G171, diamond sphero-conical indenter, F = 10, 20 and 30 N	Scratch resistance improvement by heat treatment at 621 °C for 4 h	[49]
TiC/20–60 NiMo (Ni:Mo = 4:1, 2:1, 1:1) Cr_3_C_2_/10–30 Ni WC/6–20 Co	LPS	d_TiC_ = 0.9–2.2 d_Cr_3_C_2__ = 4–6 d_WC_ = 0.9–7.4	TiC/NiMo 810–1650 Cr_3_C_2_/Ni 780–1330 WC/Co 880–1380	TRS: TiC/NiMo 730–2450 Cr_3_C_2_/Ni 670–910 WC/Co 1370–2500	Modified ASTM B611, block-on-ring (Al_2_O_3_ grinding wheel), F = 20 N	At equal HV marked difference in wear rate WC/Co outperforms cermets	[46]
Cr_3_C_2_/10–30 Ni	LPS RS	d_Cr_3_C_2__ = 4 … 6	LPS: 920–1420 RS: 890–1450	LPS: 9.5–18.0 RS: 9.8–18.5	Modified ASTM B611, block-on-ring, (Al_2_O_3_ grinding wheel), F = 20 N	RS grades outperform LPS grades	[48]
TiC/20–40 NiMo (Ni:Mo = 4:1, 2:1, 1:1) Cr_3_C_2_/10 Ni Cr_3_C_2_/40 Ni	LPS	-	810–1650 [43,44]	10.4–22.9 [43,44]	Oxidation-abrasion wear tester, abrasive: SiO_2_ (0.2 … 0.3 mm) or SiC (1 … 2 mm), T = 20, 400, 700, 900 °C, time: 5 h	The best performance at high NiMo fraction and Ni:Mo = 1:1 at ≥700 °C Cr_3_C_2_/Ni cermets outperform TiC/NiMo at ≥700 °C	[43,44,45]

* wt%, unless otherwise stated (UOS). ** LPS—liquid phase sintering; SHS–combustion synthesis; MI—melt infiltration; sinter/HIP (one-cycle LPS + post HIP); RS—reactive sintering. *** average ceramic grain size d (µm), UOS. **** Vickers hardness HV, UOS. ***** fracture toughness K_IC_ (MPa m^1/2^), UOS; TRS—transverse rupture strength (MPa). ****** room temperature, UOS.

**Table 2:** Summary of composition, processing, structural and mechanical characteristics and three-body abrasive wear testing conditions of cermets.

Composition *	Processing **	Structure ***	Mechanical Characteristics		Wear Testing Conditions ******	Key Observations	Ref.
			Hardness ****	Toughness *****			
TiC or TiB_2_/10–70 vol% Fe40Al (Fe_3_Al_2_)	LPS (1450 °C) MI (≤30 vol% Fe40Al)	Coarse grains d_TiC_ ~6	~84 HRA (30 vol% Fe40Al)	18.0 (30% Fe40Al) 13.0 (20% Fe40Al)	ASTM G65, block-on-wheel, abrasive: SiO_2_	WC-based hardmetals outperform cermets (at similar % binder)	[56]
TiC/12 vol% NiMo WC/12 vol% Co Cr_3_C_2_/12 or 33 vol% Ni	LPS	d = 2–4, (depending on composition)	-	-	ASTM G65, block-on-wheel, abrasive: SiO_2_ (0.2–0.3 mm) F = 130, 195 and 490 N	Mechanically mixed layer formation during abrasion	[60]
TiC/20–40 FeNi TiC/20–50 NiMo (Ni:Mo 4:1, 2:1) WC/10–20 Co	LPS Sinter/HIP	-	TiC/FeNi 1050–1450 TiC/NiMo 1000–1400 WC/Co 1000–1350	TRS: TiC/FeNi 1500–2400 TiC/NiMo 1700–2200 WC/Co 2300–3100	Modified ASTM G65, block-on ring, abrasive: SiO_2_ (0.1–0.2 mm), F = 3 N	Superiority of WC/Co over cermets TiC/FeNi superiority over TiC/NiMo	[37,62]
TiC/20–40 FeNi	LPS Sinter/HIP	d_TiC_ = 2.0–2.2	88.7–91.3 HRA	TRS: 1400–2300	Modified ASTM G65, block-on-ring: abrasive SiO_2_ (0.1–0.2 mm), F = 3 N	No effect of sinter/HIP on abrasion resistance	[63]
TiC/50 FeMn (13% Mn, 0.55% C)	LPS (1420 °C) HP (1400 °C, *p* = 40 MPa) SPS (1300 °C, *p* = 40 MPa, 5 min) MS (1200 °C)	d_TiC_ = 3.6–4.7	LPS 86 HRA HP 87 HRA SPS 87 HRA MS 87 HRA	TRS: LPS 1105 HP 1119 SPS 1050 MS 1230	Modified ASTM G105, block-on-ring, abrasive: SiC (~0.25 mm), F = 196 N	The lowest wear rate of MS cermets with lowest grain size	[54]
Ti(C,N)-based commercial cermet	LPS	d_TiCN_ = 0.5–2	2200	-	ASTM G105/ASTM B611, abrasives: SiO_2_, Al_2_O_3_, SiC, F = 225 N	WC/Co (1500 HV) outperforms cermet (SiC and Al_2_O_3_ abrasives)	[55]
Ti(C,N)/(12.5 Ni, 11 Mo) + 10 WC	LPS (Ford Motor Company)	d_TiCN_ = 1–4	1500–1800	7–11	Modified ASTM B611, block-on-wheel, abrasives: SiO_2_ (75–124 µm), SiC (75–88 µm), diamond (1 µm), F = 10.9 N (SiC), 5 N (SiO_2_)	Wear mechanism of cermets and WC/Co similar TiC_0.4_N_0.6_-based outperforms TiC-based cermet	[57]
TiC/20–60 wt% NiMo (Ni:Mo 4:1, 2:1, 1:1) TiC/20–40 wt% FeNi Cr_3_C_2_/10–30 wt% Ni	LPS One-cycle sinter/HIP Two-cycle sinter + HIP	d_TiC_ = 2 d_Cr_3_C_2__ = 4 … 6	TiC-based 750–1650 Cr_3_C_2_-based 700–1400	TRS: 700–2600	Modified ASTM B611, block-on-wheel, abrasive: SiO_2_ (0.1–0.3 mm), F = 40 and 200 N	Positive effect of sinter/HIP on TRS and limited to abrasion resistance of TiC-cermets	[58]
TiC/20–60 wt% NiMo (Ni:Mo 4:1, 2:1, 1:1) Cr_3_C_2_/10–30 wt% Ni WC/6–20 wt% Co	LPS	-	TiC/NiMo 810–1650 Cr_3_C_2_/Ni 780–1330 WC/Co 890–1580	TiC/NiMo 10.4–22.9 Cr_3_C_2_/Ni 7.9–14.5 WC/Co 14.7–37.3	Modified ASTM B611, block-on-wheel, abrasive: SiO_2_ (0.1–0.3 mm), F = 40 and 200 N	Wear rate of composites with equal hardness or binder vol% differs several times; WC/Co outperforms cermets	[59]
TiC/20–60 NiMo (Ni:Mo 4:1, 2:1, 1:1) WC/15 Co (reference)	LPS	d_TiC_ = 1–2	810–1650	10.4 ≥ 22.9	Modified ASTM B611, block-on-wheel, abrasive: SiO_2_ (0.1–0.3 mm), F = 40 and 200 N	Abrasion mechanism of hardmetal and cermets depends on H_a_/H_m_ ratio. Ratio Ni:Mo 2:1 is recommendable for high wear performance	[61]
TiC/20–50 NiMo (Ni:Mo 4:1, 2:1) TiC/20–40 FeNi (5, 8, 14, 17 Ni in binder WC/10–20 Co	LPS	d_TiC_ = 1.9–2.2 d_WC_ = 1.0–2.2	TiC/NiMo 890–1430 TiC/FeNi 1000–1520 WC/Co 1030–1500	TRS: TiC/NiMo 1090–1680 TiC/FeNi 1380–2450 WC/Co 1900–3000	Modified ASTM B611, block-on ring, abrasive: SiO_2_ (0.1–0.3 mm)	Cermets with suitable composition compete with WC-Co (at equal hardness)	[64]

* wt%, unless otherwise stated (UOS). ** LPS—liquid phase sintering; SPS—spark plasma sintering; HP—hot pressing; MI—melt infiltration; HIP—hot isostatic pressing; sinter/HIP (one-cycle LPS + post HIP); MS—microwave sintering. *** average ceramic grain size d (µm), UOS. **** Vickers hardness HV, UOS. ***** fracture toughness K_IC_ (MPa m^1/2^), UOS; TRS—transverse rupture strength (MPa). ****** room temperature, UOS.

**Table 3:** Summary of composition, processing, structural and mechanical characteristics and solid-particle erosive wear testing conditions of cermets.

Composition *	Processing **	Structure ***	Mechanical Characteristics		Wear Testing Conditions ******	Key Observations	Ref.
			Hardness ****	Toughness *****			
TiC/WC/NbC/18 NiMo TiC/NbC/WC/32 NiMo WC/10–25 Co WC/6 NiCo	LPS	d_Carbide_ = 1–10	-	-	ASTM G76, abrasive: SiO_2_ (75–200 µm), V = 60 m/s, α = 30, 60, 90°	TiC-NiMo cermets are at a disadvantage in relation to WC-based hardmetals	[33]
TiC/50 vol% Fe	SHS/pseudoHIP	d_TiC_ = 2–5	670	-	Modified ASTM G76, abrasives: SiO_2_ and Al_2_O_3_, V = 60 m/s, α = 30 and 90°, T = 20 and 350 °C	Brittle erosion mechanism both at room and elevated temperatures	[66]
TiC_0.7_N_0.3_/10 WC/NbC/TaC/20 Ni	LPS (1510 °C)	-	990–1250	13.4–18.3	Modified ASTM G76, abrasive: SiC (66 µm), abrasive flow: 2.33 g/s, α = 30, 60, 90°	Similar erosion behavior of ceramics and cermets WC addition favor wear resistance	[67]
Commercial cutting materials TiCN/WC/TaC/NbC/Mo_2_C/15 CoNi Al_2_O_3_, Si_3_N_4_-based ceramics	LPS (cermets)	-	Cermets 1470–1620 Ceramics 1370–1800	-	ASTM G76, abrasive: Al_2_O_3_ (70 µm), abrasive flow 2.0 g/min, α = 20 and 90°	Ceramics outperform cermets No positive effect of TaC/NbC/Mo_2_C additions	[68,69]
TiC/20 vol% Fe40Al TiB_2_/60 vol% Fe40Al WC/20 vol% Fe40Al WC/6 Co (different grain size)	MI (1450 °C) (cermets)	d_WC_ = 0.55–1.51	TiC/FeAl 1028 TiB_2_/FeAl 496 WC/FeAl 942 WC/Co 1525–1753	-	Modified ASTM G76/ASTM G211, abrasive: Al_2_O_3_ (50 µm), V = 40 m/s, α = 75°, T = 25, 180, 500, 700 °C	TiC- and TiB_2_-based cermets outperform WC-Co at > 500 °C	[87]
TiC/20–60 FeSi, FeCrSi, FeNi and FeCrNi TiC/20–30 NiMo WC-9–20 Co (different d_WC_)	LPS	TiC/Fe alloy TiC/NiMo d_TiC_ = 2–2.7 WC/Co fine, medium, coarse	TiC/Fe alloy 1050–1470 TiC/NiMo 1360–1520 WC/Co 1030–1380	TRS: TiC/Fe alloy 700–2280 TiC/NiMo 900–1300 WC/Co 1850–2950	Centrifugal accelerator, abrasive: SiO_2_ (0.2–0.3 mm), V = 80 m/s, α = 30°	Wear resistance depends on combined effect of resistance to penetration and cutting	[34]
TiC/20–40 FeNi TiC/20–50 NiMo (Ni:Mo 2:1, 4:1) WC/10–20 Co	LPS	d_TiC_ = 2–2.3	TiC/FeNi 1100–1440 TiC/NiMo 1000–1400 WC/Co 980–1350	TRS: TiC/FeNi 1400–2400 TiC/NiMo 1700–2200 WC/Co 2400–3000	Centrifugal accelerator, abrasive: SiO_2_ (0.1–0.2 mm), V = 80 m/s, α = 30°	WC/Co outperforms TiC-based cermets; TiC/FeNi outperforms TiC/NiMo (room temperature)	[37]
Cr_3_C_2_/10–20 Ni	LPS RS	d_Cr_3_C_2__ = 4–6	LPS 920–1420 RS 990–1450	LPS 9.5–18.0 RS 9.8–18.5	Centrifugal accelerator, abrasive: SiO_2_ (0.1–0.3 mm), V = 60 and 80 m/s, α = 30, 45, 60, 75 90°	RS grades outperform LPS grades	[48]
TiC/20–60 NiMo (Ni:Mo 4:1, 2:1, 1:1) TiC/20–40 FeNi Cr_3_C_2_/10–30 Ni	LPS One-cycle Sinter/HIP Two-cycle sinter + HIP	d_TiC_~2 d_Cr_3_C_2__~4–6	TiC-based 750–1650 Cr_3_C_2_-based 700–1400	TRS: 700–2600	Centrifugal accelerator, abrasive: SiO_2_ (0.1–0.3 mm), V = 80 m/s, α = 30°	Two-cycle sinter + HIP is at disadvantage over one-cycle sinter/HIP	[58]
TiC12 vol% NiMo Cr_3_C_2_/12 or 30 vol% Ni WC/12 vol% Co	LPS	d = 2 … 4 (depending on composition)	-	-	Centrifugal accelerator, abrasive: SiO_2_ (0.2–0.3 mm), V = 20 and 80 m/s, α = 30 and 90°, T = 23 and 600 °C	Mechanically mixed layer formation is an essential feature of material wear response	[60]
TiC/20–50 NiMo (Ni:Mo 4:1, 2:1) TiC/20–40 FeNi (5, 8, 14, 17 wt% Ni in binder) WC/10–20 Co	LPS	d_TiC_ = 1.9–2.2 d_WC_ = 1.0–2.2	TiC/NiMo 890–1430 TiC/FeNi 1000–1520 WC/Co 1030–1500	TRS: TiC/NiMo 1090–1680 TiC/FeNi 1380–2450 WC/Co 1900–3000	Centrifugal accelerator, abrasive: SiO_2_ (0.1–0.3 mm), V = 80 m/s, α = 30°	WC/Co outperforms cermets (at equal HV) The different wear response of WC- and TiC-based composites	[64]
TiC/40 NiMo (Ni:Mo 1:1, 2:1, 4:1)	LPS (1480 °C)	-	1068–1330	17.5–18.2	Centrifugal accelerator, abrasive: SiO_2_ (0.1–0.3 mm), V = 60 m/s, α = 75°	The erosion rate is influenced by the stress state of the, rate is lower for cermets with lower residual stresses	[70]
TiC/20–60 NiMo (Ni:Mo 1:1, 2:1, 4:1)	LPS (1400–1480 °C)	d_TiC_ = 1–5	810–1650	TRS: 730–2450	Centrifugal accelerator, abrasive: SiO_2_ (0.1–0.3 mm), V = 50 m/s, α = 30 and 90°, T = 20, 350 and 650 °C	Erosion resistance is the highest with Ni:Mo = 1:1 No significant erosion rate increase at 650 °C	[71]
TiC/20–40 NiMo TiC/20–40 FeNi Cr_3_C_2_/10–30 Ni WC/8–20 Co	LPS	d_Carbide_ = 2–6	TiC/NiMo 990–1378 TiC/FeNi 1060–1440 Cr_3_C_2_/Ni 980–1460 WC/Co 1030–1350	TiC/NiMo 11.5–18.5 TiC/FeNi 14.0–15.5 Cr_3_C_2_/Ni 9.5–18.3 WC/Co 13.0–19.0	Centrifugal accelerator, abrasives: SiO_2_ (0.1–0.3 mm), SiC (0.1–0.3 mm), V = 60 m/s, α = 75°	Modulus of elasticity may be used for evaluation of mild erosion	[72,76]
TiC/12 vol% NiMo Cr_3_C_2_/12 vol% Ni WC/12 vol% Co	LPS	d_Carbide_ = 2–4	-	-	Centrifugal accelerator, abrasive: SiO_2_ (0.1–0.3 mm), V = 20 and 80 m/s, α = 30 and 90°, T = 23 and 600 °C	Erosion depends on thickness and hardness of mechanically mixed layer The highest wear performance of TiC/NiMo at 600 °C	[73]
TiC/12 vol% NiMo Cr_3_C_2_/12 vol% Ni WC/12 vol% Co	LPS	d_Carbide_ = 1–4	~1380	TiC/NiMo 11.5 Cr_3_C_2_/Ni 9.8 WC/Co 13.0	Centrifugal accelerator, abrasive: SiC (0.1–0.3 mm), V = 60 m/s, α = 60°	Materials with high thermal conductivity possess higher erosion resistance	[74]
TiC/20 NiMo TiC/20 FeNi Cr_3_C_2_/20 Ni WC/20 Co	LPS	d_Carbide_ = 1–4	1030–1410	9.8–19.0	Centrifugal accelerator, abrasives: SiO_2_ (0.1–0.3 mm), SiC (0.1–0.3 mm), V = 20, 30, 45, 60, 80 m/s, α = 30, 45, 60, 75, 90°	Maximal erosion rate at α = 60–90°, depending on composition	[75]
TiC/20, 40 NiMo TiC/25, 40 FeNi Cr_3_C_2_/15, 30 Ni WC/8, 20 Co	LPS	d_Carbide_ = 2–6	TiC/NiMo 990, 1378 TiC/FeNi 1000, 1320 Cr_3_C_2_/Ni 980, 1410 WC/Co 1030, 1350	TiC/NiMo 11.5, 18.5 TiC/FeNi 15.0, 15.5 Cr_3_C_2_/Ni 9.8, 18.3 WC/Co 13.0, 19.0	Centrifugal accelerator, abrasives: SiO_2_ (0.1–0.3 mm), SiC (0.1–0.3 mm), V = 45 m/s, α = 60°	Materials with high thermal conductivity possess higher erosion resistance Relative ranking of composites depends on microstructure rather than on mechanical properties	[77,81]
TiC/30–50 NiMo (Ni:Mo = 2:1) TiC/30–40 FeNi Cr_3_C_2_/10–30 NiMo (Ni:Mo = 2:1) WC/8–15 Co Tool steels	LPS	-	TiC/NiMo 1000–1420 TiC/FeNi 1100–1360 Cr_3_C_2_/NiMo 1110–1420 WC/Co 960–1430	-	Centrifugal accelerator, abrasive: SiO_2_ (0.1–0.3 mm), V = 80 m/s, α = 30°	WC/Co outperforms cermets Erosion performance depends on composite stiffness	[78]
TiC/FeNi WC/10–15 Co	LPS	d_WC_ ≤ 1.0–2.2 d_TiC_ ~ 2.0	87.3–91.3 HRA	12.5–18.0	Centrifugal accelerator, abrasive: SiO_2_ (0.1–0.2 mm), V = 80 m/s, α = 30°	Erosion resistance depends on elastic modulus and proof stress	[79]
TiC/20–50 NiMo (Ni:Mo 4:1, 2:1) TiC/20–40 FeNi	LPS	d_TiC_ ~ 3	TiC/NiMo 890–1430 TiC/FeNi 1060–1445	TiC/NiMo 12.1–22.9 TiC/FeNi 13.2–15.5	Centrifugal accelerator, abrasive: SiO_2_ (0.1–0.3 mm), V = 46 and 80 m/s, α = 30, 45, 60, 75, 90°	Mechanical properties do not enable prognosis of erosion resistance	[80]
TiC/20, 40 NiMo (Ni:Mo = 2:1) TiC/40, 60 FeCrSi Cr_3_C_2_/15, 30 Ni WC/8, 15 Co	LPS	d_Carbide_ = 2–2.7	TiC/NiMo 1190–1378 TiC/FeCrSi 1150, 1360 Cr_3_C_2_/Ni 980, 1410 WC/Co 1200, 1350	-	Centrifugal accelerator, abrasives: SiO_2_ (0.1–0.3 mm) and/or SiC (0.1–0.3 mm), V = 31, 46, 61, 80 m/s, α = 30, 45, 60, 75, 90°	Maximal erosion rate at α = 60–90° depending on composition. The main wear mechanism: low-cycle fatigue Erosion resistance depends on modulus of elasticity WC/Co outperforms cermets.	[82,83,84]
TiC/40–60 FeSi WC/15 Co	LPS	-	TiC/FeSi 1020–1360 WC/Co 1200	-	Centrifugal accelerator, abrasives: Al_2_O_3_ (90 µm), glass spheres (650 µm), V = 30 and 80 m/s, α = 67°	Erosion mechanism depends on H_a_/H_m_ Ductile response of TiC/FeSi and WC/Co	[85]
Cr_3_C_2_/20 Ni + Mo/Cu additions	LPS (1250–1300 °C)	d_Cr_3_C_2__ = 3–15 (depending on composition)	1010–1220	10.1–10.4	Centrifugal accelerator, abrasive: SiO_2_ (0.1–0.3 mm), V = 31 and 80 m/s, α = 30 and 75°	Mo addition and low residual stresses enhance wear resistance.	[86]

* wt%, unless otherwise stated (UOS). ** LPS—liquid phase sintering; SHS—combustion synthesis; MI—melt infiltration; HIP—hot isostatic pressing; sinter/HIP (one-cycle LPS + post HIP); RS—reactive sintering. *** average ceramic grain size d (µm), UOS. **** Vickers hardness HV, UOS. ***** fracture toughness K_IC_ (MPa m^1/2^), UOS; TRS—transverse rupture strength (MPa). ****** room temperature, UOS.

**Table 4:** Erosion rate of carbide composites at 600 °C [73].

Grade	Materials Composition	Erosion Rate, mm^3^/kg			
		Impact Angle, 30°		Impact Angle, 90°	
		Velocity, 20 m/s	Velocity, 80 m/s	Velocity, 20 m/s	Velocity, 80 m/s
WC	WC-12vol%Co	9.07	88.4	10.6	59.8
CC	Cr_3_C_2_-12vol%Ni	0.3	5.2	0.7	20.5
TC	TiC-12vol%NiMo	0.2	2.2	0.2	5.9

**Table 5:** Summary of composition, processing, structural and mechanical characteristics and slurry erosion testing conditions of cermets.

Composition *	Processing **	Structure ***	Mechanical Characteristics		Wear Testing Conditions ******	Key Observations	Ref.
			Hardness ****	Toughness *****			
TiC_0.7_N_0.3_/15 Ni + Mo_2_C/WC/TaC/NbC additions	Sinter/HIP (1450 °C, *p* = 5 MPa) or LPS (1440 °C)	- [90] d_TiCN_ = 1.16–1.43 [95] d_TiCN_ = 0.66–1.68 [102]	1116–1796 [90] 1274–1530 [95] 91.2–94.0 HRA [102]	5.3–10.1 [90] TRS: 1663–1716 [95] TRS: 928–1351 [102]	Slurry-pot test, Al_2_O_3_ (5 wt%, 150–250 µm) slurry: neutral (distilled water) saline (NaCl solution) acidic (H_2_SO_4_ solution)	Mo_2_C additions dramatically increase erosion resistance	[90,95,102]
TiC_0.7_N_0.3_/10Mo_2_C 15Ni	LPS (1440 °C) or Sinter/HIP (1450 °C, *p* = 5 MPa)	- [91] d_TiCN_ = 1.16 [92] - [101]	92.6 HRA [91] 1530 [92] 92.6 HRA [101]	11.5 [91] TRS: 1716 [92] TRS 1650 [101]	Slurry-pot test, Al_2_O_3_ (1 or 5 wt%, 150–250 µm) slurry: - neutral (distilled water) - saline (NaCl solution) - acidic (H_2_SO_4_ solution) Slurry of SiO_2_ (5%, 0.1–0.3 mm) - saline (seawater)	Binder loss is the primary degradation mode	[91,92,101]
TiC_0.7_N_0.3_/10Mo_2_C Cr_3_C_2_ 15Ni (1, 3, 5, 7 Cr_3_C_2_)	LPS (1440 °C) and/or Sinter/HIP (1450 °C, *p* = 5 MPa)	- [93] d_TiCN_ = 0.72–1.58 d_TiCN_ = 0.81–1.58 [98] - [100]	93.5 HRA [93] 1574–1817 [97] 92.6–93.2 HRA [98] 92.6–93.5 HRA [100]	10.8 [93] 9.5–12.9 [97] TRS: 1300–1380 [98] TRS: 1300–1490 [100]	Slurry-pot test, Al_2_O_3_ (5 wt%, 150–250 µm) slurry: neutral (distilled water) acidic (H_2_SO_4_ solution) alkaline (NaOH solution)	Erosion performance is improved by Cr_3_C_2_ additions in all environments	[93,97,98,100]
TiC_0.7_N_0.3_/10Mo_2_C 15Ni/Co (different Ni/Co ratios)	Sinter/HIP (1450 °C, *p* = 5 MPa)	-	92.0–92.5 HRA	TRS: 1510–1650	Slurry pot test, SiO_2_ (5 wt%, 0.1–0.3 mm) slurry: - neutral (distilled water) - saline (seawater)	Erosion resistance improvement with the addition of Co	[94]
Ti(C, N)/10Mo_2_C 15Ni (different TiC/TiN ratios)	LPS (1440 °C)	d_TiCN_ = 0.78–1.44	92.2–92.5 HRA	-	Slurry pot test, Al_2_O_3_ (5 wt% 150–250 µm) slurry: - alkaline (NaOH solution) - acidic (H_2_SO_4_ solution)	The best performance of TiC_0.9_N_0.1_-based cermets in alkaline and acidic conditions	[96,99]
TiC_0.96_/33–60 FeCr (0–25 Cr in binder) WC/15 Co	LPS	-	TiC-FeCr 1030–1430 WC-Co 1200	-	Slurry pot test, SiO_2_ (5 wt%, 0.3–0.4 mm) slurry: - neutral (water) - alkaline (NaOH)	Corrosion proof cermets outperform WC/Co in neutral environment	[103]
Cr_3_C_2_/10–40 Ni	LPS	d_Cr_3_C_2__ = 2–5	900–1490	9.5–19.0	Slurry-jet impingement test, SiO_2_ (6, 8, 10 wt%, ~0.1 mm) slurry: V = 4 m/s, α = 90°: - saline (seawater) T = 20–40 °C	Erosion performance depends on the interplay of binder fraction and the abrasive concentration	[104,105]

* wt%, unless otherwise stated (UOS). ** LPS—liquid phase sintering; sinter/HIP (one-cycle LPS + post HIP). *** average ceramic grain size d (µm), UOS. **** Vickers hardness HV, UOS. ***** fracture toughness K_IC_ (MPa m^1/2^), UOS; TRS—transverse rupture strength (MPa). ****** room temperature, UOS.

**Table 6:** Working conditions (abrasion or erosion, mechanical loads, temperature and corrosion) and recommendations for ceramic–metal composite selection.

Working Conditions			Recommendations for Ceramic–Metal Composite Selection
Type of Wear	Temperature, °C	Mechanical Loads	
Abrasive wear (two- or three-body abrasion)	Low temperature ≤ 500 °C	Low	WC-Co (<20 vol%) TiC-Fe alloy (mild abrasion)
		High	WC-Co (>20 vol%)
	High temperature > 500 °C	low	Ti(C,N)/TiC-NiMo (<20 vol%) Cr_3_C_2_-Ni (<20 vol%, at ≥700 °C)
		High	Ti(C,N)/TiC-NiMo (>20 vol%)
Solid-particle erosion	low temperature ≤ 500 °C	Low	WC-Co (<20 vol%) Ti(C,N)/TiC-based cermets (<20 vol%) (mild erosion)
		High	WC-Co (>20 vol%)
	High temperature > 500 °C	Low	Ti(C,N)/TiC-NiMo (<20 vol%) Cr_3_C_2_-Ni (<20 vol%, at ≥600 °C)
		High	Ti(C,N)/TiC-NiMo (>20 vol%)
Slurry erosion (erosion-corrosion)	Low temperature	Low	Cr_3_C_2_-Ni (<20 vol%) Corrosion-resistant grades of Ti(C,N)/TiC-cermets (<20 vol% of binder)

## Data Availability

Not applicable.
